# Systematic Analysis of an Invasion-Related 3-Gene Signature and Its Validation as a Prognostic Model for Pancreatic Cancer

**DOI:** 10.3389/fonc.2021.759586

**Published:** 2021-12-15

**Authors:** Dafeng Xu, Yu Wang, Yuliang Zhang, Zhehao Liu, Yonghai Chen, Jinfang Zheng

**Affiliations:** ^1^ Department of Hepatobiliary and Pancreatic Surgery, Hainan General Hospital, Hainan Affiliated Hospital of Hainan Medical University, Haikou, China; ^2^ Geriatric Medicine Center, Hainan General Hospital, Hainan Affiliated Hospital of Hainan Medical University, Haikou, China; ^3^ Department of Otolaryngology Head and Neck Surgery, Hainan General Hospital, Hainan Affiliated Hospital of Hainan Medical University, Haikou, China

**Keywords:** Pancreatic adenocarcinoma (PAAD), invasive-related genes, LY6D, BCAT1, ITGB6, prognosis

## Abstract

**Background:**

Pancreatic adenocarcinoma (PAAD) is a malignant tumor of the digestive system that is associated with a poor prognosis in patients owing to its rapid progression and high invasiveness.

**Methods:**

Ninety-seven invasive-related genes obtained from the CancerSEA database were clustered to obtain the molecular subtype of pancreatic cancer based on the RNA-sequencing (RNA-seq) data of The Cancer Genome Atlas (TCGA). The differentially expressed genes (DEGs) between subtypes were obtained using the limma package in R, and the multi-gene risk model based on DEGs was constructed by Lasso regression analysis. Independent datasets GSE57495 and GSE62452 were used to validate the prognostic value of the risk model. To further explore the expression of the hub genes, immunohistochemistry was performed on PAAD tissues obtained from a large cohort.

**Results:**

The TCGA-PAAD samples were divided into two subtypes based on the expression of the invasion-related genes: C1 and C2. Most genes were overexpressed in the C1 subtype. The C1 subtype was mainly enriched in tumor-related signaling pathways, and the prognosis of patients with the C1 subtype was significantly worse than those with the C2 subtype. A 3-gene signature consisting of *LY6D*, *BCAT1*, and *ITGB6* based on 538 DEGs between both subtypes serves as a stable prognostic marker in patients with pancreatic cancer across multiple cohorts. *LY6D*, *BCAT1*, and *ITGB6* were over-expressed in 120 PAAD samples compared to normal samples.

**Conclusions:**

The constructed 3-gene signature can be used as a molecular marker to assess the prognostic risk in patients with PAAD.

## Background

Pancreatic adenocarcinoma (PAAD) is a malignant tumor of the digestive tract and is the fourth leading cause of cancer-related deaths worldwide (1). Since the early symptoms of PAAD are not obvious, its diagnosis is often difficult, and the primary tumor exhibits vascular invasion. Approximately 80-85% of patients with pancreatic cancer present with distant metastases at the time of diagnosis, thus making radical resection ineffective (2). Therefore, the search for more accurate and effective diagnostic and prognostic markers is of great significance for the stratification and individualization of patients with pancreatic cancer in the clinical setting.

At present, the prognostic prediction of patients with pancreatic cancer is mainly based on clinicopathologic features. However, the prognosis of patients with the same clinical stage and grade differs because of the high heterogeneity of pancreatic cancer. Moreover, the malignant progression of pancreatic cancer is accompanied by genetic changes. Therefore, the study of the molecular mechanisms underlying pancreatic cancer progression is key to prolonging the overall survival of patients with pancreatic cancer (3). However, the effect of a single gene in predicting the prognosis of a pancreatic tumor is often unsatisfactory and presents with some limitations; the combined detection of multiple genes is expected to facilitate the prognostic prediction of patients with pancreatic cancer. With the rapid development of bioinformatics and sequencing technology, an increasing number of studies have provided potential prognostic assessments for patients with pancreatic cancer. Li et al. (4) constructed a 9-gene signature using macrophage phenotypic switch-related genes in patients with pancreatic cancer. Wang et al. (5) constructed a 9-gene signature for predicting PAAD based on the expression of immune-related genes. However, most prognostic models include a large number of genes, which greatly increases the cost of medical treatment in clinical practice. Moreover, most studies are based on a comprehensive analysis of public databases and lack experimental data to verify and explore the role of the identified genes in the development of pancreatic cancer.

In this study, a molecular subtype of pancreatic cancer was constructed based on invasion-related genes using gene expression data from The Cancer Genome Atlas (TCGA), Gene Omnibus Expression (GEO), and other public databases. The relation between molecular subtypes, prognosis, and clinical features was further analyzed. A 3-gene prognostic model, composed of *LY6D*, *BCAT1*, and *ITGB6*, constructed with differentially expressed genes (DEGs) between the PAAD subtypes, could be used to evaluate the prognosis of patients with PAAD.

## Materials And Methods

### Data Source and Preprocessing

RNA-sequencing (RNA-seq) data and clinical follow-up information data from TCGA-PAAD samples were downloaded from the TCGA database. The expression data and clinical information from the GSE57495, GSE62452 and GSE28735 datasets were downloaded from the GEO database. A total of 97 invasion-related genes were collected from the CancerSEA website (**Supplement Table 1**).

The RNA-seq data from the TCGA-PAAD dataset was processed through the following steps: 1) Samples with no clinical follow-up information were removed; 2) The ENSEMBL gene IDs were converted to the Gene Symbol format; 3) The median value was calculated with multiple Gene Symbol expressions.

The following steps were used to process the GEO dataset: 1) Samples without clinical follow-up information were removed; 2) The probe IDs were converted to the Gene Symbol format; 3) Probes that corresponded to multiple genes were removed. 4) When multiple probes correspond to one gene, take the average value as the gene expression.

After preprocessing, we enrolled 176 samples from TCGA-PAAD, 63 samples from GSE57495 data set, 66 samples from GSE62452 data set, and 42 samples from GSE28735 dataset. The clinical characteristics of the patient samples are listed in **Table 1**.

**Table 1:** Clinical characteristics of patient samples.

Clinical Features	TCGA-PAAD	GSE57495	GSE62452	GSE28735
**OS**				
0	84	21	16	13
1	92	42	50	29
**T Stage**				
T1	7			
T2	24			
T3	140			
T4	3			
TX	2			
**N Stage**				
N0	49			
N1	122			
NX	5			
**M Stage**				
M0	79			
M1	4			
MX	93			
**Stage**				
I	21			
II	145			
III	3			
IV	4			
X	3			
**Grade**				
G1	30			
G2	94			
G3	48			
G4	2			
GX	2			
**Gender**				
Male	96			
Female	80			
**Age**				
≤65	93			
>65	83			
**Alcohol**				
YES	100			
NO	64			
Unknown	12			
**Chemotherapy**				
YES	116			
NO	60			
**Radiation therapy**				
YES	32			
NO	101			
Unknown	43			

### Consistency Clustering Algorithm and Gene Set Enrichment Analysis (GSEA)

The expression profiles of 97 invasion-related genes were extracted from the TCGA-PAAD dataset, and univariate Cox regression analysis was performed to select significant prognostic genes using coxph function in R (*p* < 0.05). Next, the genes with significant results from the univariate Cox analysis were clustered using ConsensusClusterPlus (V1.48.0; parameters: reps = 100, pitem = 0.8, pfeature = 1, and distance = “Canberra”). The Pam and Canberra distances were used as a clustering algorithm and distance measure, respectively.

The gene set c2.cp.kegg.v7.0.symbols.gmt was selected, and significantly enriched pathways between different molecular subtypes were analyzed by GSEA. PAAD samples were divided into either a C1 or C2 subtype based on gene expression data from the TCGA-PAAD dataset in the GSEA input file. The thresholds for pathway enrichment analysis were *p* < 0.05 and false discovery rate (FDR) < 0.25.

### Identification of DEGs

DEGs between C1 and C2 subtypes were calculated using the limma package (6), and the filtering thresholds were FDR < 0.05 and | log 2 fold-change (FC) | > 1. The identified DEGs were subjected to Kyoto Encyclopedia of Genes and Genomes (KEGG) pathway analysis and Gene Ontology (GO) enrichment analysis using the WebGestaltR (v0.4.2) package in R software.

### Construction of a Risk Model Based on Invasion-Related Genes

#### Random Grouping of Training Set Samples

The 176 samples in the TCGA-PAAD dataset were divided into a training set and validation set. To avoid the effect of random assignment bias on the stability of subsequent modeling, 200 samples were assigned to random groups. The samples were grouped according to a training set: validation set ratio of 3:2. After dividing the samples, there were 106 samples in the training set and 70 samples in the validation set.

### Lasso Regression Analysis and Stepwise Regression Analysis of Training Set Data

Univariate Cox regression analysis was performed for each DEG (538 in total) using the coxph function in R to identify prognostic genes, and *p* < 0.05 was selected as the threshold for filtering. Lasso regression analysis was performed to further reduce the number of genes in the risk model using the glmnet package in R (7). In stepwise regression analysis, the selection of the model starts with the most complex model from which one variable is removed at a time to reduce the number of parameters according to the Akaike Information Criterion (AIC). The smaller the p-value of the regression model, the more superior the model. This indicates that the regression model fits the data well with fewer parameters. The prognostic model is made fit for clinical applications by performing stepwise regression to further reduce the number of genes.

The prognostic model was constructed based on the following equation:


$$\text{risk score}=\sum_{i=1}^{n}\beta \text{i}\times \exp({\text{G}}_{\text{i}})$$

where n refers to the number of genes identified for the multivariate Cox regression model; exp(Gi) is the expression value of gene i; and βi is the coefficient for gene i.

### Immunohistochemistry

To verify the expression of the candidate three genes, tissue microarrays (TMA) comprised of 120 PAAD tissues and 30 normal samples were obtained from Shanghai Outdo Biotech Co., Ltd. (Shanghai, China). The clinicopathological details of 120 PAAD tissues were shown in **Table 2**. The studies were conducted in accordance with the International Ethical Guidelines for Biomedical Research Involving Human Subjects (CIOMS), and the research protocols were approved by the Ethics Committee of Hainan General Hospital, Hainan Affiliated Hospital of Hainan Medical University.

**Table 2:** The clinicopathological details of 120 PAAD tissues.

Clinical Features	PAAD-IHC
**T Stage**	
T1	4
T2	30
T3	61
T4	1
TX	24
**N Stage**	
N0	54
N1	63
NX	3
**M Stage**	
M0	112
M1	8
MX	0
**Stage**	
I	21
II	90
III	1
IV	8
X	0
**Grade**	
G1	1
G2	76
G3	38
G4	0
GX	5
**Gender**	
Male	66
Female	54
**Age**	
≤65	77
>65	43

The TMA slides were dried overnight at 37°C, dewaxed in xylene, and dehydrated in a gradient ethanol series. Antigens retrieval was performed by heating the tissue sections in a microwave oven inside a vessel filled with EDTA antigen retrieval buffer (pH 9.0). Subsequently, the tissue sections were immersed in 3% hydrogen peroxide for 25 min to block the activity of endogenous peroxides. Next, the TMA tissues were coated with 3% bovine serum albumin (BSA) and sealed at room temperature for 30 min to reduce non-specific staining. Then, the TMA slides were incubated with anti-LY6D (1: 200 dilution; Novus Biologicals, NBP1-84029), anti-BCAT1 (1:50 dilution; Abcam, ab197941), and anti-ITGB6 (1:10 dilution; Abcam, ab197672) overnight at 4°C.

The tissues were rinsed with 0.01 mol/L phosphate buffer saline (PBS; pH = 7.4) for 5 min each. The tissues were incubated at room temperature for 50 min with horseradish peroxidase (HRP)-labeled goat anti-rabbit secondary antibody (1:200 dilution, ServiceBio, GB23303). Then, the tissues were washed in PBS and stained with 3,3-diaminobenzidine (DAB). Finally, the TMA sections were counterstained with Mayer’s hematoxylin, dehydrated, and fixed. To evaluate IHC staining, semi-quantitative scoring criteria were used.

The stained sections were scored by three pathologists who were blinded to the patients’ clinical characteristics. The scoring system was based on the proportion of positively stained cells in all tissues and the staining intensity of these positively stained cells. The staining intensity was classified as follows: 0 (negative), 1 (weak), 2 (moderate), or 3 (strong). The staining ratio of positive cells was classified as follows: 0 (<5%), 1 (5%-25%), 2 (26%-50%), 3 (51%-75%), or 4 (> 75%). According to the staining intensity and the proportion of positively stained cells, the tissues were graded as follows: 0-1 grade, negative (-); > 1-4, weakly positive (+); > 4-8, moderately positive (++), and > 8- 12, strongly positive (+++).

## Results

### Identification of Molecular Subtypes Based on Invasion-Related Genes

**Figure 1:**
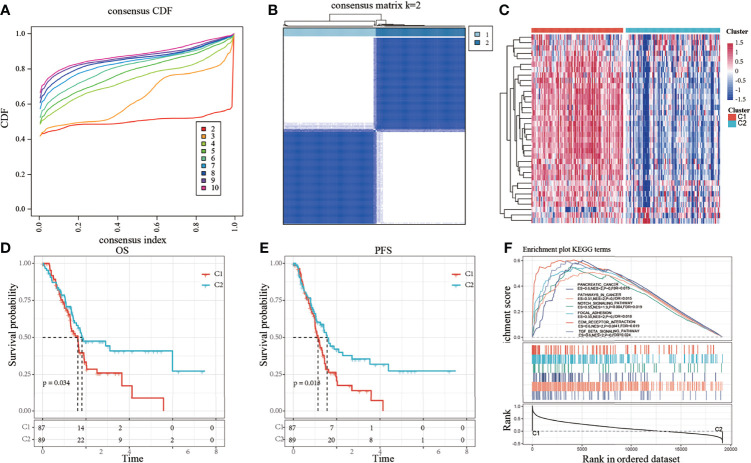
**(A)** Cumulative distribution function (CDF); **(B)** Consistent clustering heatmap when k = 2; **(C)** Cluster heatmap of 35 prognostic genes; **(D)** Overall survival (OS) curve based on molecular subtypes in all samples of The Cancer Genome Atlas-pancreatic adenocarcinoma (TCGA-PAAD) dataset; **(E)** Progression-free survival (PFS) curve based on molecular subtypes in all TCGA-PAAD samples; **(F)** Involvement of tumor-related pathways between molecular subtypes of the TCGA dataset.

Thirty-five genes were found to be significantly associated with the prognosis of pancreatic cancer using univariate Cox analysis (**Supplement Table 2**). Consistent cluster analysis showed that the samples could be clustered together at k=2 (**Figures 1A, B**). The expression levels of the invasion-related genes were significantly different between the C1 and C2 subtypes, and most genes were overexpressed in the C1 subtype (**Figure 1C**). The relationship between the subtypes and prognosis was further analyzed, and results showed that there were significant differences in survival times between the C1 and C2 subtypes (**Figures 1D, E**, log-rank *p* < 0.05).

The results of the GSEA analysis showed the activation of more tumor-related pathways in the C1 subtype, such as pathways in cancer, notch signaling pathway, focal adhesion, extracellular matrix (ECM)-receptor interaction, and TGF-β signaling pathway (**Figure 1F**), suggesting that the C1 subtype is more closely related to cancer than the C2 subtype.

### Analysis of DEGs Between Subtypes

According to the thresholds mentioned in the methods section, 538 DEGs were obtained, of which 531 genes were upregulated and 7 genes were downregulated (**Supplement Table 3**). The results demonstrated that the C1 subtype contains more upregulated genes than the C2 subtype. The volcano map of upregulated and downregulated DEGs between the two subtypes is shown in **Supplementary Figure 1A**. The expression patterns of the top 50 upregulated DEGs and all the downregulated DEGs were shown in a heatmap (**Supplementary Figure 1B**). The results of the GO enrichment analysis of DEGs showed that 548 Biological Process (BP) terms were significantly different between the two subtypes (FDR < 0.05). The first 15 BP terms were plotted (FDR < 0.05), as shown in **Supplementary Figure 1C**. The first 15 Cellular Component (CC) terms were plotted, as shown in **Supplementary Figure 1D**. Fifty-two Molecular Function (MF) terms were significantly different between the two subtypes (FDR < 0.05). The results of the first 15 MF terms are shown in **Supplementary Figure 1E**. The KEGG pathway analysis of DEGs showed 27 significantly enriched pathways (FDR < 0.05). Further visualization of the top 10 enriched pathways showed that genes were significantly enriched in tumor-related pathways such as the ECM-receptor interaction pathway, focal adhesion, and the PI3K-Akt signaling pathway (**Supplementary Figure 1F**).

### Comparison of Immune Score Between Molecular Subtypes

**Figure 2:**
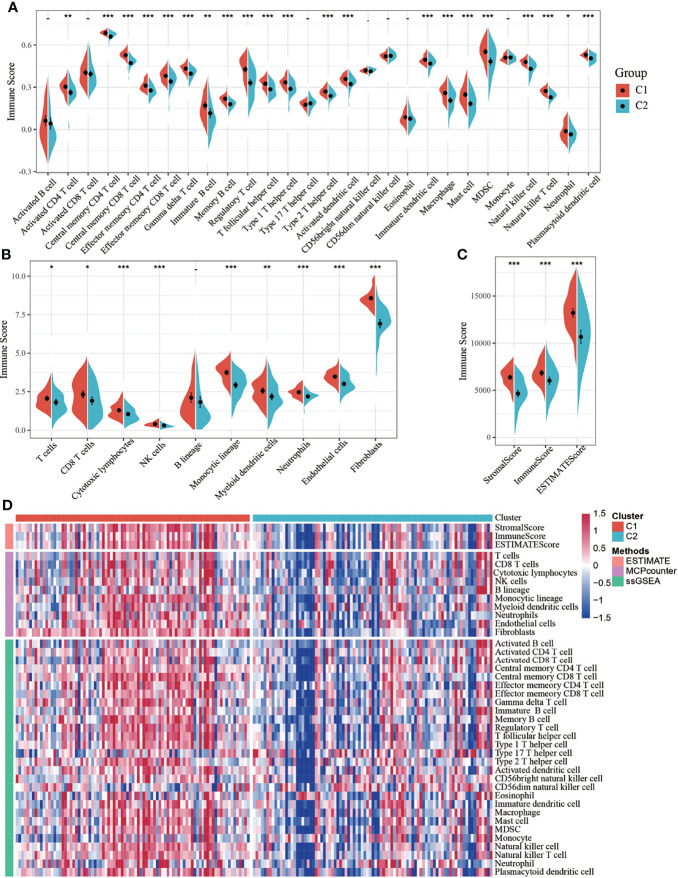
**(A)** Comparison of single-sample gene set enrichment analysis (ssGSEA) immune scores between molecular subtypes in all samples of The Cancer Genome Atlas-pancreatic adenocarcinoma (TCGA-PAAD) dataset; **(B)** Comparison of MCPcounter immune scores between molecular subtypes of the TCGA dataset; **(C)** Comparison of estimated immune scores between molecular subtypes of the TCGA dataset; **(D)** Heat map comparing three software immune scores among molecular subtypes of the TCGA dataset. *P < 0.05; **P < 0.01; ***P < 0.001.

To identify the relationship between molecular subtypes and immune scores in the TCGA-PADD dataset, the ESTIMATE package was used to evaluate the three immune scores: stromal, immune, and estimate scores. MCPcounter was used to evaluate 10 types of immune cells, and the single-sample GSEA (ssGSEA) method in the GSCA package was used to evaluate 28 types of immune cells (8). Meanwhile, the difference in immune scores between the two molecular subtypes was compared. The results showed that the immune scores of the C1 subtype were higher than those of the C2 subtype (**Figures 2A**–**C**). The heatmap of the immune scores of the two subtypes is shown in **Figure 2D**.

### Risk Model of Pancreatic Cancer Based on Invasion-Related Genes

By performing univariate Cox analysis of the DEGs between the C1 and C2 subtypes, 18 prognostic genes were identified. Lasso regression analysis was performed to further reduce the number of prognostic genes. The locus of each independent variable is shown in **Supplementary Figure 2A**. As the value of lambda (λ) increased, the number of independent variables tending to zero also increased. A 10-fold cross-validation was performed to construct the model, and the confidence interval under each λ is shown in **Supplementary Figure 2B**. The model was found to be optimal when λ = 0.05667557, so a λ of 0.0567557 was chosen for further analysis of the prognostic genes. Six genes, namely *LY6D*, *DKK1*, *BICC1*, *BCAT1*, *ITGB6*, and *PTGES* were identified as the hub genes when λ = 0.0567557. The number of model genes was further reduced by stepwise regression, and finally, three genes were obtained: *LY6D*, *BCAT1*, and *ITGB6*. The risk score based on the final 3-gene prognostic model was calculated as follows: Risk score = 0.1627483 * *LY6D* + 0.2210480 * *BCAT1* + 0.2005339 * *ITGB6*.

**Figure 3:**
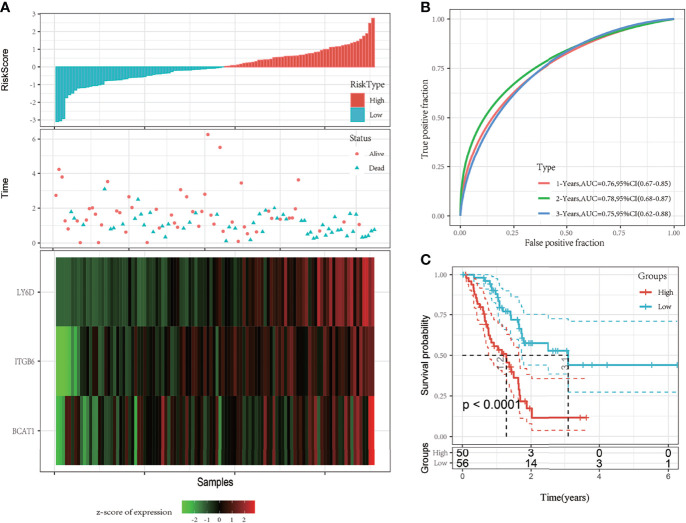
**(A)** The risk score, survival time and state, and expression of the 3-gene signature were studied in The Cancer Genome Atlas (TCGA) training set. **(B)** Receiver operating characteristic (ROC) curve and area under the curve (AUC) of the 3-gene signature; **(C)** The Kaplan-Meier (KM) survival curve distribution of the 3-gene signature in the training set.

Risk scores of each sample were calculated based on the expression level of *LY6D*, *BCAT1*, and *ITGB6*, and a risk score distribution was plotted for each sample, as shown in **Figure 3A**. The results showed that a higher risk score was associated with worse outcomes, and high expression levels of *LY6D*, *BCAT1*, and *ITGB6* were associated with a higher risk score. The timeROC package was used to analyze the receiver operating characteristic (ROC) curve of risk score; the 1-, 2-, and 3-year predictive classification efficiencies were 0.76, 0.78, and 0.75, respectively, as shown in **Figure 3B**. The samples were divided into a high-risk group and a low-risk group based on the risk scores. Finally, 50 and 56 samples were placed into the high- and low-risk groups, respectively. The KM curve showed a significant difference in the expression of DEGs between the high- and low-risk groups (*p* < 0.01) (**Figure 3C**).

### Verification of Robustness of the 3-Gene Prognostic Model Using Internal and External Datasets

#### Verification of the Robustness of the 3-Gene Prognostic Model Using Internal Datasets

**Figure 4:**
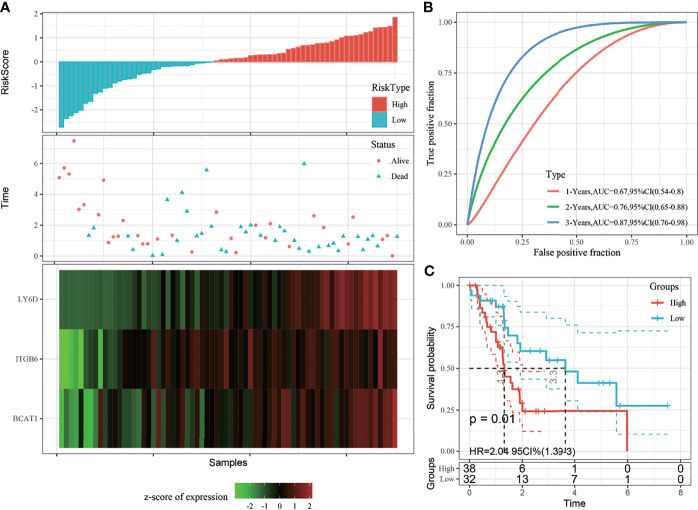
**(A)** Risk score, survival time, survival status, and 3-gene signature expression in The Cancer Genome Atlas (TCGA) training set; **(B)** ROC curve and area under the curve (AUC) of the 3-gene signature; **(C)** Distribution of the Kaplan-Meier (KM) survival curve of the 3-gene signature in the TCGA validation set.

To determine the robustness of the model, the risk score distribution of the TCGA validation set and all dataset samples was calculated using the same coefficients as those of the training set. The risk score distribution of the TCGA validation set suggested that samples with a high risk score are associated with a worse prognosis, as shown in **Figure 4A**. The 1-, 2-, and 3-year predictive classification efficiencies of the risk scores were 0.67, 0.76, and 0.87, respectively (**Figure 4B**). These results demonstrated that the prognosis of the high-risk group was significantly worse than that of the low-risk group (**Figure 4C**).

**Figure 5:**
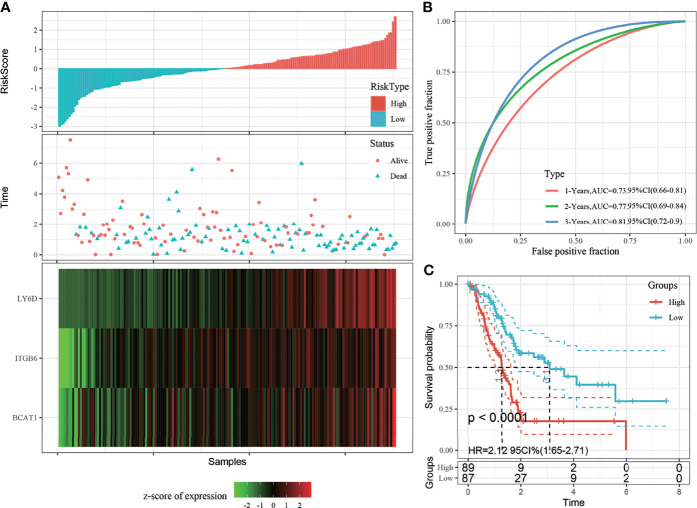
**(A)** Risk score, survival time, and 3-gene signature expression in all The Cancer Genome Atlas (TCGA) datasets; **(B)** Receiver operating characteristic (ROC) curve and area under the curve (AUC) of the 3-gene signature; **(C)** Distribution of the Kaplan-Meier (KM) survival curve of 3-gene signature in all TCGA datasets.

The risk score distribution trend of all TCGA datasets was consistent with those of the training set (**Figure 5A**). The predictive classification efficiencies of the 1-, 2-, and 3-year ROCs were 0.73, 0.77, and 0.81, respectively (**Figure 5B**). According to the above classification, 89 and 87 samples were categorized into the high- and low-risk groups, respectively, in all TGGA datasets. The prognosis of the high-risk group was significantly worse than that of the low-risk group (**Figure 5C**).

#### Validation of the Robustness of the 3-Gene Prognostic Model Using Three Independent Cohorts

**Figure 6:**
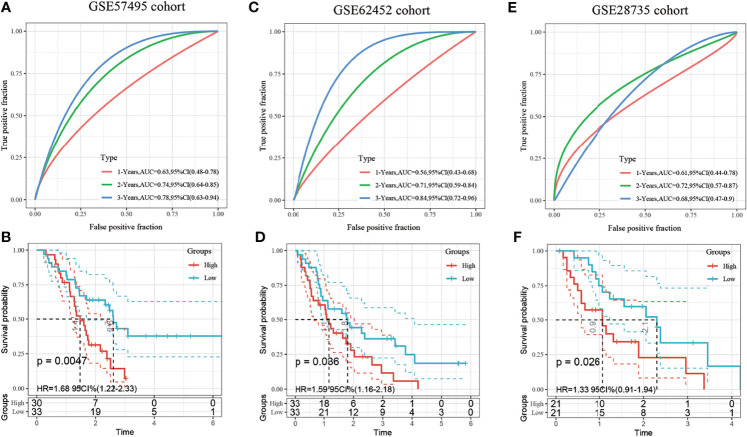
**(A)** Receiver operating characteristic (ROC) curve and area under the curve (AUC) of the 3-gene signature; **(B)** Distribution of the Kaplan-Meier (KM) survival curve of the 3-gene signature in the GSE57495 dataset; **(C)** Receiver operating characteristic (ROC) curve and area under the curve (AUC) of the 3-gene signature; **(D)** Distribution of the Kaplan-Meier (KM) survival curve of the 3-gene signature in the GSE62452 independent validation set; **(E)** Receiver operating characteristic (ROC) curve and area under the curve (AUC) of the 3-gene signature; **(F)** Distribution of the Kaplan-Meier (KM) survival curve of the 3-gene signature in the GSE28735 independent validation set.

The robustness of the model was further verified with three independent validation cohorts GSE57495, GSE62452 and GSE28735. The 1-, 3-, and 5-year ROCs in the GSE57495 dataset were 0.63, 0.74, and 0.78, respectively (**Figure 6A**). The 1-, 3-, and 5-year ROCs in the GSE62452 were 0.56, 0.71, and 0.84, respectively (**Figure 6C**). The 1-, 3-, and 5-year ROCs in the GSE28735 were 0.61, 0.72, and 0.68, respectively (**Figure 6E**). Therefore, the predictive performance of the model was stable in different cohorts. Finally, the samples with a risk score greater than zero after zscore method were classified into the high-risk group and those with a risk score less than 0 were classified into the low-risk group. In the GSE57495 cohort, 30 and 33 samples were categorized into the high- and low-risk groups, respectively, with significant prognostic differences between the two groups (**Figure 6B**). In the GSE62452 cohort, 33 samples each were categorized into the high and low-risk groups, respectively, with significant prognostic differences between the two groups (**Figure 6D**). In the GSE28735 cohort, 21 samples each were categorized into the high and low-risk groups, respectively, with significant prognostic differences between the two groups (**Figure 6F**).

### Risk Model and Prognostic Analysis of Clinical Features

**Figure 7:**
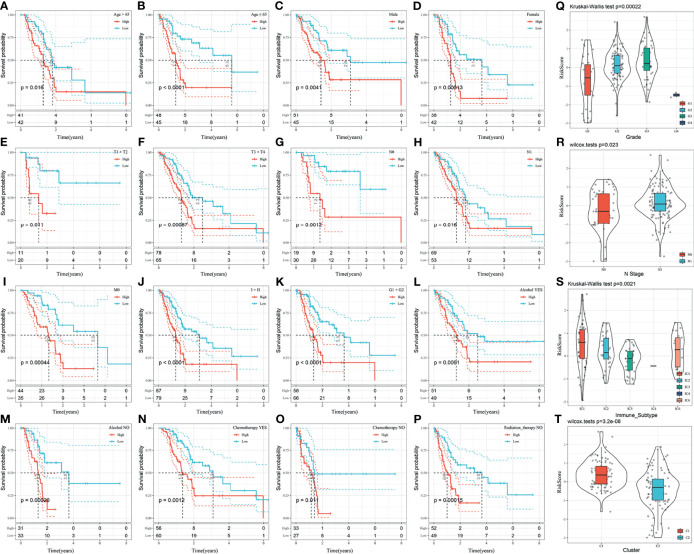
**(A–P)** Performance of the risk model in distinguishing different clinical characteristics of patients; **(Q)** Comparison of the risk score between the samples grouped according to the tumor grade; **(R)** Comparison of the risk score comparison between the samples grouped according to the N-Stage; **(S)** Comparison of the risk score in existing immune molecular subtypes between grouped samples; **(T)** Comparison of the risk score between samples of the molecular subtypes identified in this study.

Further analysis of the relationship between the risk score and clinical features showed that the 3-gene prognostic model could significantly distinguish between age, sex, TNM stage, clinical stage, tumor grade, alcohol consumption, chemotherapy, and radiation therapy between the high- and low-risk groups (**Figures 7A**–**P**, *p* < 0.05). This suggests that the model also has good predictive power in distinguishing different clinical features.

The distribution of the risk score among the clinical features of the two groups was further compared. The results demonstrated that the risk score is significantly different between the N-stage and tumor grade (*p* < 0.05). The higher the tumor grade, the higher the risk score (**Figure 7Q**). The risk score of N1 was significantly higher than that of N0 (**Figure 7R**). The risk score of the C1 subtype with a poor prognosis was significantly higher than that of the C2 subtype with a good prognosis (**Figure 7T**). Moreover, the risk score was significantly different among existing immune molecular subtypes (**Figure 7S**).

### Construction of the Nomogram

**Figure 8:**
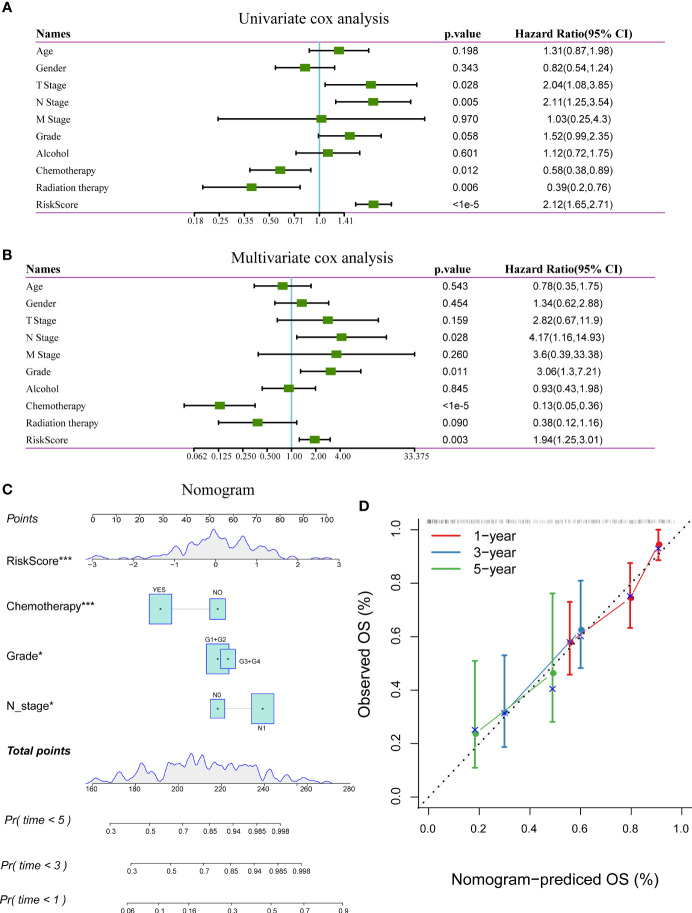
**(A)** Results of univariate analysis of clinical characteristics and risk scores; **(B)** Results of multivariate analysis clinical characteristics and risk scores; **(C)** Nomogram based on clinical characteristics and risk scores; **(D)** Nomogram for predicting survival rate of patients with pancreatic cancer along with correction factors. *P < 0.05; ***P < 0.001.

In the TCGA-PAAD dataset, the univariate Cox regression analysis showed a significant correlation between the risk type and survival, while the multivariate Cox regression analysis showed a significant correlation between the risk score (Hazard ratio [HR] = 1.94, 95% confidence interval [CI] = 1.25–3.01, and *p* = 0.003) and survival. These results demonstrate the good predictive performance of the identified 3-gene prognostic model in clinical applications. Furthermore, the N stage (HR = 4.17, 95% 1.16–14.93, and *p* = 0.028) and grade (HR = 3.06, 95% CI = 1.3–7.21, and *p* = 0.011) were identified as independent prognostic risk factors for patients with pancreatic cancer. Chemotherapy (HR = 0.13, 5% CI = 0.05–0.36, and *p* < 0.001) was identified as an independent prognostic protective factor (**Figures 8A, B**).

The nomogram, which displays the results of the risk model directly and effectively, can be conveniently applied to the prediction of an outcome. The nomogram uses the length of the line to indicate the degree of influence that different variables have on the result and the influence of different values of variables on the result. According to the results of the univariate and multivariate analyses, the nomogram was constructed with the following clinical features: N stage, tumor grade, chemotherapy, and risk score (**Figure 8C**). The results showed that the risk score has the greatest effect on survival prediction, indicating that the risk model based on the 3-gene signature can accurately predict the prognosis of patients with pancreatic cancer. A calibration diagram was used to visualize the nomogram. The results showed that the nomogram performed well in determining the prognostic risk of patients with pancreatic cancer (**Figure 8D**).

### Comparison of Risk Model With Other Models

**Figure 9:**
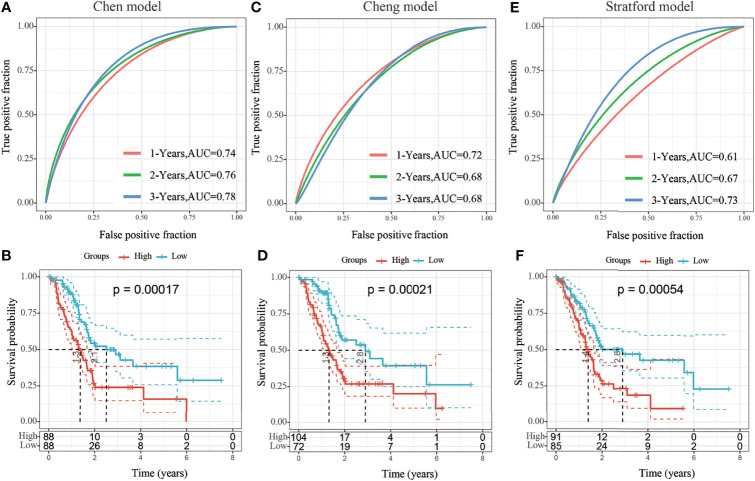
**(A, B)** Receiver operating characteristic (ROC) curve of the 15-gene signature (Chen) risk model and KM curve of High/Low-risk samples; **(C, D)** ROC of the 7-gene signature (Cheng) risk model and the Kaplan-Meier (KM) curve of samples from high- and low-risk groups; **(E, F)** ROC of 6-gene signature (Stratford) risk model and the KM curve of samples from high- and low-risk groups.

Four prognostic risk models, including 15-gene signature (Chen) (9), 7-gene signature (Cheng) (10), and 6-gene signature (Stratford) (11) models, were compared with the identified 3-gene prognostic model. To facilitate comparison among the models, the risk score of each TCGA-PAAD sample was calculated using the same method, and the risk score was zscored according to the corresponding gene in all three models. Genes with a risk score greater than zero were categorized into a high-risk group and those with a risk score less than zero were categorized into a low-risk group. The prognosis difference between the two groups was further analyzed. There were significant differences in outcomes between the high-risk and low-risk groups in all three risk models (**Figures 9B, D, F**, log-rank *p* < 0.05), the area under the curve (AUC)s at 1-, 2-, and 3-year of Cheng and Stratford models were lower than that of our model (**Figures 9C, E**). Although our 1-year AUC is smaller than the Chen model (0.73 vs 0.74), the AUC at 2 and 3 years is larger than his (0.77 vs 0.76, 0.81 vs 0.78, respectively) (**Figure 9A**) (**Supplement Table 4**). Therefore, the 3-gene signature identified in this study represents a more reasonable and efficient model to determine the prognostic risk of patients with pancreatic cancer with the use of fewer genes.

### Expression of LY6D, BCAT1, and ITGB6 in Pancreatic Cancer

**Figure 10:**
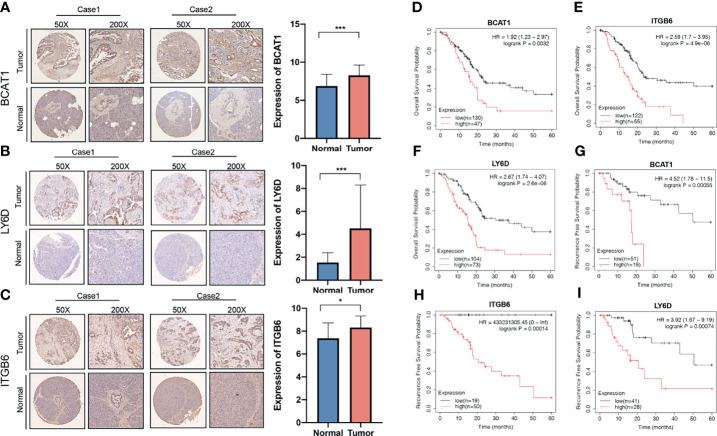
Association of the expression of invasion-related genes with prognosis of pancreatic cancer. Expression of **(A)** *BCAT1*, **(B)** *LY6D*, and **(C)** *ITGB6* genes in pancreatic cancer and normal tissues. The relationship between the expressions of **(D)** *BCAT1*, **(E)** *LY6D*, and **(F)** *ITGB6* genes with overall survival. The relationship between the expressions of **(G)** *BCAT1*, **(H)** *LY6D*, and **(I)** *ITGB6* genes with recurrence-free survival. ***p < 0.001, *p < 0.05.

The differences in the expression of the *LY6D*, *BCAT1*, and *ITGB6* genes in PAAD and adjacent tissues were investigated. The expressions of *LY6D*, *BCAT1*, and *ITGB6* in 120 cases of pancreatic cancer and 30 cases of para-carcinoma were detected by immunohistochemistry. The results showed that *BCAT1*, *LY6D*, and *ITGB6* were significantly overexpressed in cancer tissues (**Figures 10A**–**C**). Many cases in the TMA cohort were not effectively followed up. Therefore, to compensate for this limitation, the Kaplan-Meier plotter database was used to obtain 177 samples with overall survival data and 69 cases with recurrence-free survival data. The results showed that patients with high expression of *LY6D*, *BCAT1*, and *ITGB6* genes have a significantly worse prognosis than those with a low expression both in terms of overall survival and recurrence-free survival (**Figures 10D**–**I**). Our immunohistochemical results demonstrated that LY6D, BCAT1, and ITGB6 proteins were all overexpressed in PAAD samples compared to normal samples. Therefore, it can be speculated that these genes act as oncogenes in pancreatic cancer, and the upregulation of these genes is associated with a significantly worse prognosis in patients with pancreatic cancer.

### Flow Chart of Research Methodology

**Figure 11:**
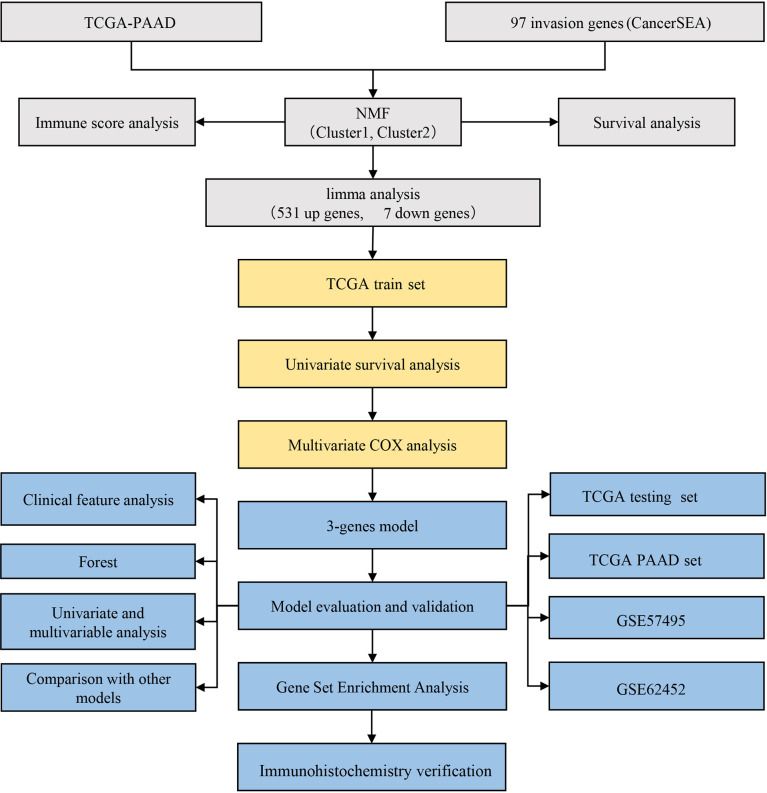
Flow chart of research methodology.

A flowchart has been drawn to allow readers to better understand the research process of this study (**Figure 11**).

## Discussion

Pancreatic cancer is a highly aggressive malignancy that is associated with a high mortality rate and poor prognosis. The 5-year survival rate for patients with pancreatic cancer is less than 10% (12). In 2021, 60,430 new pancreatic cancer cases and 48,220 pancreatic cancer-related deaths are expected in the United States (12). By 2030, pancreatic cancer is estimated to be the second most common cause of cancer-related deaths in the United States (13). The malignant progression of pancreatic cancer is often accompanied by changes in the expression of multiple genes, and the abnormal expression of specific genes may affect the prognosis of patients with pancreatic cancer. These genes may also serve as effective targets for personalized cancer therapy (14, 15). In recent years, with the rapid development of sequencing technology, high-throughput genomics has allowed for the exploration of key genes involved in cancer tumorigenesis and development. Moreover, high-throughput genomics allows for further analysis of the mechanisms related to tumorigenesis and development.

In this study, 176 TCGA-PAAD samples were genotyped based on 97 invasion-related genes, and two subtypes (C1 and C2) were obtained. The C1 subtype with a poor prognosis was more associated with the involvement of tumor-related pathways such as the Notch signaling pathway and ECM-receptor interaction. The Notch signaling pathway plays an important role in the maintenance of pancreatic tumor phenotypes (16), and the downregulation of the Notch receptor is associated with decreased proliferation, increased apoptosis, anchor-dependent growth, and decreased invasiveness of pancreatic cancer cells (17). However, matrix proteins derived from tumor cells may promote the development and metastasis of ductal adenocarcinoma of the pancreas (18). Five hundred and thirty-eight DEGs between the C1 and C2 subtypes were identified using the limma package, of which 531 genes were upregulated, and 7 genes were downregulated. We constructed a 3-gene signature using the *LY6D*, *BCAT1*, and *ITGB6* genes out of the 538 identified DEGs.

Lymphocyte 6 (Ly6) complex is a group of alloantigens, and LY6D is an important member of the Ly6 family. LY6D plays an important role in the maintenance of phenotypic and transcriptome heterogeneity of progenitor cells and the proliferation and differentiation of lymphocyte B during the early stages of lymphogenesis (19, 20). LY6D also plays an important role in cancer; it serves as a prognostic marker for advanced prostate cancer (21) and stage I non-small cell lung carcinoma (NSCLC) (22), drug resistance-associated marker for laryngeal squamous cell carcinoma (23), long-range metastasis marker for patients with ESR1-positive breast cancer (24), and a marker of urothelial and squamous cell differentiation (25). Apart from its involvement in cell adhesion, LY6D also regulates important interactions between endothelial cells and head and neck squamous cell carcinoma cells (26). In addition to glucose and fatty acid metabolism, amino acid metabolism plays an important role in tumor metabolic reprogramming.

The study has shown that the metabolism of Branched-chain amino acids (BCAA) is potentially linked with development of pancreatic ductal adenocarcinoma (27), and BCAT1, an enzyme involved in the degradation of branched-chain amino acids, is responsible for initiating the catabolism of such amino acids (28).

It has been reported that pancreatic ductal adenocarcinoma cells reprogram fibroblasts to upregulate the expression of BCAT1, to meet the cancer cells’ demand for branched-chain α-ketoacid (BCKAs) under BCAA deprivation (29).

The expression of BCAT1 is also upregulated in hepatocellular carcinoma (HCC) (30), breast cancer (31), and NSCLC (32), and indicates a poor prognosis. In HCC, BCAT1 plays a pathogenic role by promoting cell proliferation and chemoresistance (33). BCAT1 regulates mTOR-mediated autophagy *via* branched-chain amino acid metabolism, thus reducing the sensitivity of cancer cells to cisplatin (34).

As a member of the integrin β (ITGB) superfamily, the overexpression of ITGB6 is associated with the upregulation of the Notch signaling pathway in pancreatic cancer and is associated with immunosuppression in pancreatic cancer (35). Nine genetic markers, including ITGB6, can be used to predict the overall survival of patients with pancreatic cancer (36). ITGB6, which is highly expressed in colorectal cancer, is associated with a poor prognosis (37). ITGB6 can also be used as a tumor-specific surface antigen (TSA) to identify cell surface targets of CAR-T cell therapy and antibody-drug conjugates in breast cancer (38). Studies have shown that ITGB6 was a liver-metastasis-related gene for PAAD patients (39) and the overexpression of ITGB6 was significantly associated with advanced AJCC stage and histologic grade, and worse prognosis in pancreatic cancer (40). Our immunohistochemical results showed that LY6D, BCAT1, and ITGB6 were all overexpressed in pancreatic cancer, which was consistent with the previous results.

Although there are many multi-gene prognostic models for PAAD, there is no model based on invasion-related gene signature to predict the prognosis of pancreatic cancer. Invasion genes play an important role in metastasis as well as the development of cancer. Moreover, some prognostic signatures contain multiple genes (15-gene signature, 7-gene signature, and 6-gene signature), indicating that it is necessary to assess the expression profile of more genes in a patient-specific manner, which adds extra cost to medical care. Our 3-gene prognostic model has a higher ROC than the above models in terms of prediction of 1-, 2-, and 3-year survival rates of patients with pancreatic cancer, while having fewer genes. Therefore, our model has certain advantages in PAAD.

However, our model also presents certain limitations. First, information in the TCGA database is primarily limited to Caucasian and African populations; therefore, and data from the Asian population are missing from this study. Additionally, our study was a retrospective study of patients with pancreatic cancer, and prospective studies should be conducted to validate the prognostic characteristic and confirm the stable performance of the 3-gene prognostic model. Finally, the molecular mechanisms by which LY6D, BCAT1, and ITGB6 drive the malignant progression of pancreatic cancer require further verification.

## Conclusions

In this study, we divided the TCGA-PAAD samples into two subtypes based on the differential expression of the invasion-related genes and constructed a prognostic molecular signature consisting of three genes, including *LY6D*, *BCAT1*, and *ITGB6*, based on the DEGs between the two subtypes. The *LY6D*, *BCAT1*, and *ITGB6* genes were upregulated in pancreatic cancer samples. The 3-gene prognostic model also exhibited a good AUC in both the training and validation sets. Therefore, this 3-gene prognostic model, based on the expression of three invasion-related genes, may be used to assess the prognosis of patients with pancreatic cancer. This will help in the stratification of patients for personalized cancer therapy.

## Data Availability Statement

The original contributions presented in the study are included in the article/**Supplementary Material**. Further inquiries can be directed to the corresponding author.

## Author Contributions

DX and YW designed the study, performed data analysis, and wrote the manuscript. YZ, ZL, and YC performed data collection. JZ supervised the manuscript. All authors contributed to the article and approved the submitted version.

## Funding

This research was supported by Natural Science Foundation of Hainan Province (820MS130); Construction Project of Hainan Provincial Clinical Medical Research Center for malignant tumors of Digestive tract system.

## Conflict of Interest

The authors declare that the research was conducted in the absence of any commercial or financial relationships that could be construed as a potential conflict of interest.

## Publisher’s Note

All claims expressed in this article are solely those of the authors and do not necessarily represent those of their affiliated organizations, or those of the publisher, the editors and the reviewers. Any product that may be evaluated in this article, or claim that may be made by its manufacturer, is not guaranteed or endorsed by the publisher.

## Supplementary Material

Supplementary Figure 1 The volcano map of upregulated and downregulated DEGs between the two subtypes.

Supplementary Figure 2
**(A)** For each independent variable, the horizontal axis represents the log value of the independent variable lambda, and the vertical axis represents the coefficient of the independent variable. **(B)** Confidence interval under each lambda.

## Abbreviations

PAAD, Pancreatic adenocarcinoma; DEGs, Differentially expressed genes; TCGA, The Cancer Genome Atlas; GEO, Gene Expression Omnibus; *GSEA, Gene Set Enrichment Analysis;* FDR, False discovery rate; AIC, Akaike Information Criterion;

KEGG, Kyoto Encyclopedia of Genes and Genomes; GO, Gene Ontology; BP, Biological Process; CC, Cellular Component; MF, Molecular Function; OS, Overall survival; CDF, Cumulative distribution function; ROC, Receiver operating characteristic.
