# Supplementary figures and images for: Systematic Analysis of an Invasion-Related 3-Gene Signature and Its Validation as a Prognostic Model for Pancreatic Cancer
Source: Front Oncol. 2021 Dec 15;11:759586. doi: 10.3389/fonc.2021.759586 (PMC8715959; doi:10.3389/fonc.2021.759586)

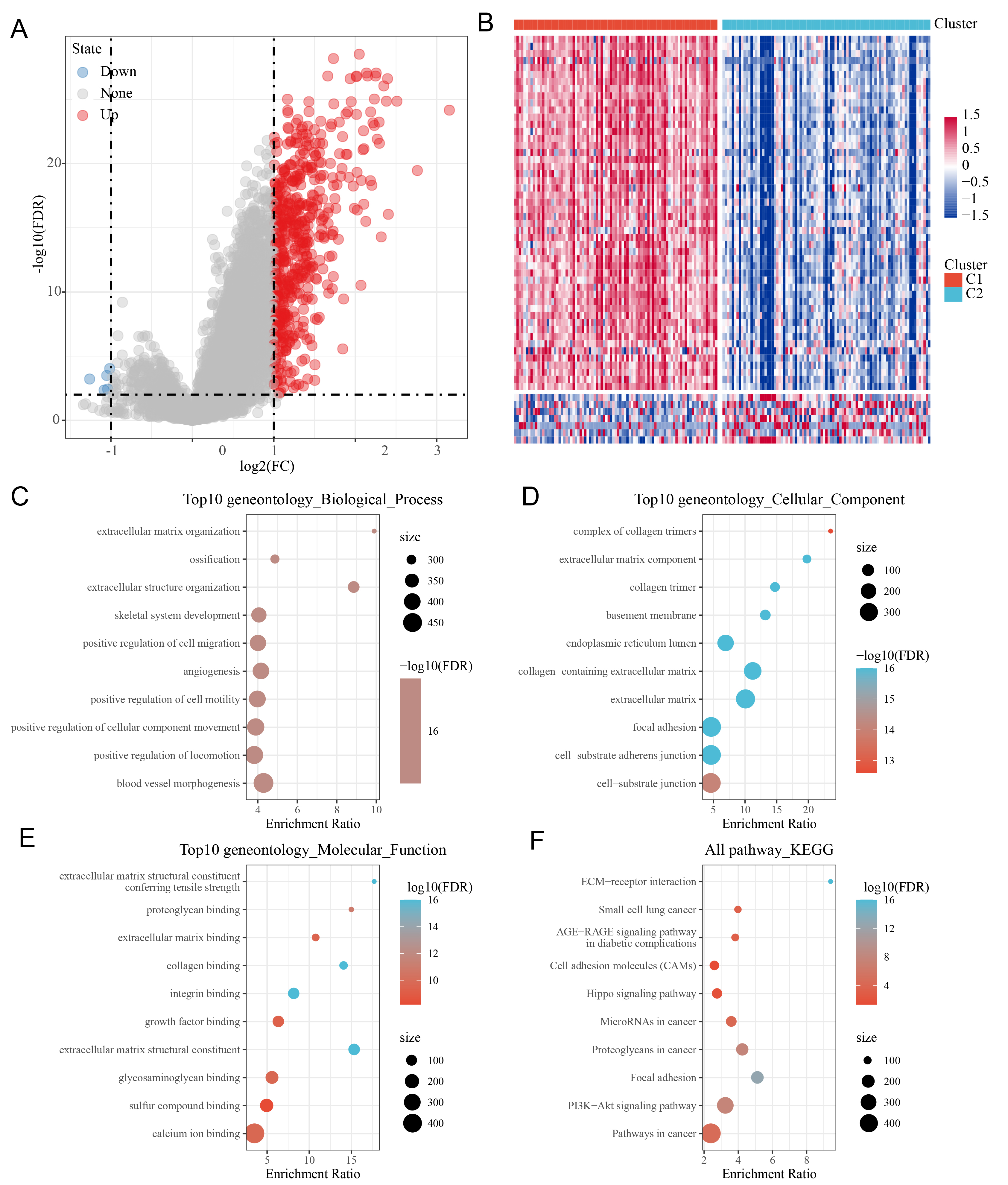

Supplement: Supplementary Figure 1 — The volcano map of upregulated and downregulated DEGs between the two subtypes. [file Image_1.jpeg]

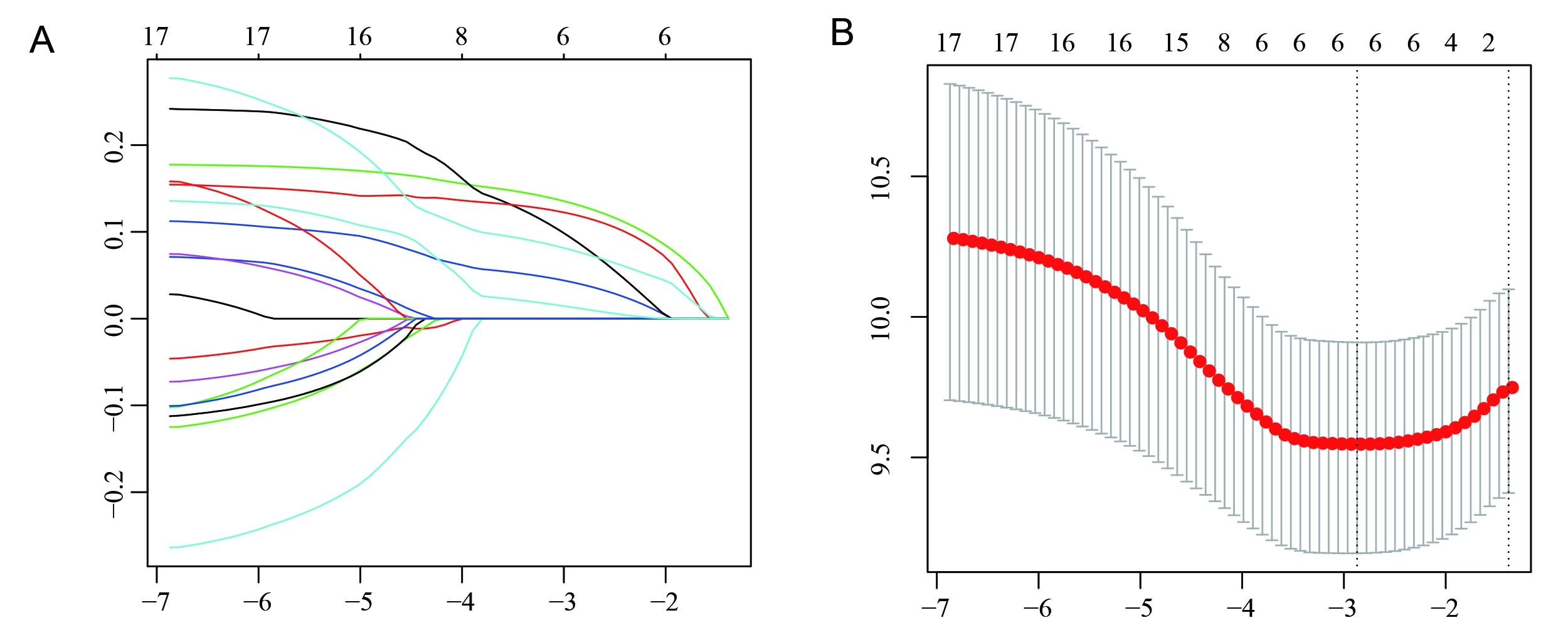

Supplement: Supplementary Figure 2 — (A) For each independent variable, the horizontal axis represents the log value of the independent variable lambda, and the vertical axis represents the coefficient of the independent variable. (B) Confidence interval under each lambda. [file Image_2.jpeg]
