# Supplementary material for: Systematic Analysis of an Invasion-Related 3-Gene Signature and Its Validation as a Prognostic Model for Pancreatic Cancer
Source: Front Oncol. 2021 Dec 15;11:759586. doi: 10.3389/fonc.2021.759586 (PMC8715959; doi:10.3389/fonc.2021.759586)
Supplement: Supplementary file 3 [file Table_1.docx]

Supplement Table 1. 97 invasion-related genes

| **Genes** | **p.value** | **HR** | **Low 95%CI** | **High 95%CI** |
| --- | --- | --- | --- | --- |
| AEBP1 | 0.36631171 | 1.00019287 | 0.999774485 | 1.000611421 |
| AKR1B1 | 0.27330894 | 0.99536844 | 0.987135356 | 1.00367019 |
| AMD1 | 0.1865278 | 1.01348728 | 0.993539607 | 1.033835458 |
| SLC25A5 | 0.81498069 | 1.000134 | 0.999012183 | 1.001257083 |
| ATP5PB | 0.3307457 | 1.00411799 | 0.995836883 | 1.012467962 |
| BAG1 | 0.88071305 | 0.9991716 | 0.98841485 | 1.010045409 |
| BGN | 0.63252599 | 1.0000577 | 0.999821226 | 1.000294224 |
| C1QB | 0.28707305 | 1.00031978 | 0.99973111 | 1.000908805 |
| CALD1 | 0.19261179 | 1.00114953 | 0.999420769 | 1.002881289 |
| CAPG | 0.01940977 | 1.00138596 | 1.000223753 | 1.002549514 |
| CCNE1 | 0.27785702 | 1.01537605 | 0.987773891 | 1.043749521 |
| CDH11 | 0.03499686 | 1.00628821 | 1.000441401 | 1.012169196 |
| CKS1B | 0.00330517 | 1.01973601 | 1.006526418 | 1.033118959 |
| CKS2 | 0.00039586 | 1.00460533 | 1.002054952 | 1.007162195 |
| COL1A1 | 0.46242629 | 1.00003007 | 0.999949876 | 1.000110265 |
| COL1A2 | 0.09648837 | 1.00011121 | 0.999980073 | 1.000242364 |
| COL3A1 | 0.08236799 | 1.00006348 | 0.999991857 | 1.000135104 |
| COL5A1 | 0.12765611 | 1.000771 | 0.99977917 | 1.001763808 |
| COL5A2 | 0.01430984 | 1.00101717 | 1.000203164 | 1.001831842 |
| COL6A2 | 0.77204587 | 1.00007718 | 0.999555108 | 1.000599535 |
| COL6A3 | 0.01716161 | 1.0010049 | 1.000178397 | 1.001832078 |
| COL10A1 | 0.00408494 | 1.00172929 | 1.000548639 | 1.002911343 |
| COL11A1 | 0.00290408 | 1.00235232 | 1.000803375 | 1.003903665 |
| COMP | 0.19306547 | 1.00079818 | 0.999596482 | 1.002001316 |
| CSE1L | 0.00125173 | 1.01196467 | 1.004680397 | 1.019301752 |
| VCAN | 0.01459986 | 1.00201817 | 1.000398156 | 1.003640813 |
| CTSK | 0.12924279 | 1.00059633 | 0.999825987 | 1.001367263 |
| DAB2 | 0.29462708 | 1.00482671 | 0.995818907 | 1.013915994 |
| DDX5 | 0.03477024 | 0.99763273 | 0.995439865 | 0.999830417 |
| EDNRA | 0.0091421 | 1.01055334 | 1.002607995 | 1.018561643 |
| FAP | 0.02295157 | 1.01227614 | 1.001687527 | 1.022976683 |
| FBN1 | 0.05567138 | 1.00326183 | 0.999921101 | 1.006613713 |
| FN1 | 0.00047315 | 1.00038362 | 1.000168504 | 1.000598787 |
| GNAS | 0.02949062 | 0.99852852 | 0.997205515 | 0.999853286 |
| H2AFZ | 0.06987833 | 1.00287319 | 0.999766941 | 1.005989097 |
| HMGB2 | 0.01862222 | 1.00568963 | 1.000948146 | 1.010453572 |
| HNRNPU | 0.0445249 | 1.00738232 | 1.000179955 | 1.01463654 |
| HSD17B4 | 0.65967495 | 1.00346096 | 0.988148309 | 1.01901091 |
| CCN1 | 0.08887338 | 1.00048177 | 0.999926795 | 1.001037058 |
| INHBA | 0.00054887 | 1.0047443 | 1.002050726 | 1.007445122 |
| LAMB1 | 0.28689585 | 1.001488 | 0.998751169 | 1.004232328 |
| LAMC1 | 0.0084872 | 1.00543082 | 1.001383944 | 1.009494044 |
| LGALS1 | 0.08954029 | 1.00023096 | 0.999964346 | 1.00049764 |
| LOX | 0.00156032 | 1.00868902 | 1.003296365 | 1.014110656 |
| LOXL2 | 0.01060948 | 1.00334483 | 1.000778288 | 1.005917947 |
| LUM | 0.12063347 | 1.00016087 | 0.999957733 | 1.000364051 |
| MMP2 | 0.07778386 | 1.00033279 | 0.999962972 | 1.00070275 |
| MMP11 | 0.32775644 | 1.00020386 | 0.999795624 | 1.000612263 |
| HNRNPM | 0.24874384 | 0.99496479 | 0.986466623 | 1.003536171 |
| NDUFB7 | 0.03356182 | 0.99841901 | 0.996963224 | 0.99987692 |
| YBX1 | 0.04994175 | 1.00058533 | 1.000000149 | 1.001170847 |
| PDGFRB | 0.10225923 | 1.00190759 | 0.99961989 | 1.004200525 |
| PLAU | 1.38E-05 | 1.00124944 | 1.000685805 | 1.001813391 |
| PRRX1 | 0.06562072 | 1.00793694 | 0.999489302 | 1.016455977 |
| PNN | 0.78568263 | 0.9988256 | 0.990401448 | 1.007321415 |
| PPIC | 0.04638982 | 1.00508372 | 1.000081126 | 1.010111344 |
| PROS1 | 0.6671563 | 1.00224161 | 0.992066077 | 1.012521512 |
| PSMA2 | 0.66693578 | 1.01608217 | 0.944872151 | 1.092658917 |
| PSMB4 | 0.97784065 | 0.99996614 | 0.997579587 | 1.002358397 |
| RGS4 | 0.05169348 | 0.98773602 | 0.975534055 | 1.000090611 |
| SNAI2 | 0.0096863 | 1.0055293 | 1.001337119 | 1.009739035 |
| SPOCK1 | 0.00129569 | 1.01265847 | 1.004927168 | 1.02044926 |
| TGFBI | 7.98E-06 | 1.00282386 | 1.001583505 | 1.004065755 |
| THBS2 | 0.0070798 | 1.00091137 | 1.000248011 | 1.001575175 |
| THY1 | 0.62619542 | 1.00053336 | 0.998388952 | 1.002682364 |
| TNFAIP6 | 0.05224266 | 1.00771267 | 0.999925452 | 1.015560542 |
| UBE2V2 | 0.10795911 | 1.02506937 | 0.994584489 | 1.05648864 |
| ADAM12 | 0.15472702 | 1.00784526 | 0.997055754 | 1.018751516 |
| MFAP5 | 0.59751225 | 1.00114887 | 0.996890673 | 1.005425266 |
| ITGBL1 | 0.12692514 | 1.00596446 | 0.998311994 | 1.013675581 |
| TP53I3 | 0.05845055 | 1.00540881 | 0.999807144 | 1.01104187 |
| NUAK1 | 0.22279922 | 1.00957242 | 0.994227332 | 1.025154351 |
| HNRNPDL | 0.63706959 | 1.0012332 | 0.996120137 | 1.006372517 |
| TXNDC9 | 0.11806174 | 1.02047283 | 0.994865503 | 1.046739279 |
| LRRC17 | 0.91811134 | 1.00085445 | 0.984690713 | 1.017283518 |
| IFI30 | 0.81084892 | 0.98367074 | 0.859595097 | 1.125655709 |
| POSTN | 0.04524187 | 1.00067423 | 1.000014283 | 1.001334603 |
| CBX1 | 0.82412273 | 0.99884131 | 0.988680881 | 1.009106156 |
| NID2 | 0.0823604 | 1.01635055 | 0.997921952 | 1.035119461 |
| RRAS2 | 0.15990052 | 1.00835651 | 0.996721699 | 1.020127139 |
| RALY | 0.05589217 | 1.00996655 | 0.999750588 | 1.020286899 |
| SEPHS2 | 0.28399361 | 1.00213286 | 0.998234529 | 1.006046411 |
| HEY1 | 0.10238053 | 0.96286936 | 0.920130913 | 1.007592925 |
| MXRA5 | 0.02486749 | 1.00217953 | 1.000275152 | 1.004087544 |
| OLFML2B | 0.9850604 | 1.00003006 | 0.996888335 | 1.003181691 |
| TMEM158 | 0.06561138 | 1.00484891 | 0.999687667 | 1.010036809 |
| WWTR1 | 0.00260248 | 1.01400491 | 1.004867015 | 1.023225906 |
| GREM1 | 0.01345915 | 1.00291715 | 1.000602967 | 1.005236677 |
| NOX4 | 0.02034163 | 1.04692582 | 1.007141771 | 1.088281407 |
| CLEC4A | 0.55178148 | 0.99135316 | 0.963399587 | 1.020117828 |
| COPZ2 | 0.85146624 | 1.00089635 | 0.991553725 | 1.010327006 |
| ASPN | 0.07094273 | 1.00142207 | 0.999878727 | 1.002967797 |
| CEMIP | 0.20464523 | 1.0026395 | 0.998563897 | 1.006731731 |
| CRISPLD2 | 0.59313846 | 1.00066665 | 0.998223338 | 1.003115937 |
| TUBB6 | 0.20292266 | 1.00353422 | 0.998099104 | 1.00899894 |
| LRRC15 | 0.07037211 | 1.00520073 | 0.999568786 | 1.010864407 |
| TUBB | 0.00420764 | 1.00158618 | 1.000499716 | 1.002673816 |
