# Supplementary material for: Systematic Analysis of an Invasion-Related 3-Gene Signature and Its Validation as a Prognostic Model for Pancreatic Cancer
Source: Front Oncol. 2021 Dec 15;11:759586. doi: 10.3389/fonc.2021.759586 (PMC8715959; doi:10.3389/fonc.2021.759586)
Supplement: Supplementary file 4 [file Table_2.docx]

Supplement Table 2. 35 prognostic genes by Univariate Cox analysis

| **Genes** | **p.value** | **HR** | **Low 95%CI** | **High 95%CI** |
| --- | --- | --- | --- | --- |
| CAPG | 0.01940977 | 1.00138596 | 1.000223753 | 1.002549514 |
| CDH11 | 0.03499686 | 1.00628821 | 1.000441401 | 1.012169196 |
| CKS1B | 0.00330517 | 1.01973601 | 1.006526418 | 1.033118959 |
| CKS2 | 0.00039586 | 1.00460533 | 1.002054952 | 1.007162195 |
| COL5A2 | 0.01430984 | 1.00101717 | 1.000203164 | 1.001831842 |
| COL6A3 | 0.01716161 | 1.0010049 | 1.000178397 | 1.001832078 |
| COL10A1 | 0.00408494 | 1.00172929 | 1.000548639 | 1.002911343 |
| COL11A1 | 0.00290408 | 1.00235232 | 1.000803375 | 1.003903665 |
| CSE1L | 0.00125173 | 1.01196467 | 1.004680397 | 1.019301752 |
| VCAN | 0.01459986 | 1.00201817 | 1.000398156 | 1.003640813 |
| DDX5 | 0.03477024 | 0.99763273 | 0.995439865 | 0.999830417 |
| EDNRA | 0.0091421 | 1.01055334 | 1.002607995 | 1.018561643 |
| FAP | 0.02295157 | 1.01227614 | 1.001687527 | 1.022976683 |
| FN1 | 0.00047315 | 1.00038362 | 1.000168504 | 1.000598787 |
| GNAS | 0.02949062 | 0.99852852 | 0.997205515 | 0.999853286 |
| HMGB2 | 0.01862222 | 1.00568963 | 1.000948146 | 1.010453572 |
| HNRNPU | 0.0445249 | 1.00738232 | 1.000179955 | 1.01463654 |
| INHBA | 0.00054887 | 1.0047443 | 1.002050726 | 1.007445122 |
| LAMC1 | 0.0084872 | 1.00543082 | 1.001383944 | 1.009494044 |
| LOX | 0.00156032 | 1.00868902 | 1.003296365 | 1.014110656 |
| LOXL2 | 0.01060948 | 1.00334483 | 1.000778288 | 1.005917947 |
| NDUFB7 | 0.03356182 | 0.99841901 | 0.996963224 | 0.99987692 |
| YBX1 | 0.04994175 | 1.00058533 | 1.000000149 | 1.001170847 |
| PLAU | 1.38E-05 | 1.00124944 | 1.000685805 | 1.001813391 |
| PPIC | 0.04638982 | 1.00508372 | 1.000081126 | 1.010111344 |
| SNAI2 | 0.0096863 | 1.0055293 | 1.001337119 | 1.009739035 |
| SPOCK1 | 0.00129569 | 1.01265847 | 1.004927168 | 1.02044926 |
| TGFBI | 7.98E-06 | 1.00282386 | 1.001583505 | 1.004065755 |
| THBS2 | 0.0070798 | 1.00091137 | 1.000248011 | 1.001575175 |
| POSTN | 0.04524187 | 1.00067423 | 1.000014283 | 1.001334603 |
| MXRA5 | 0.02486749 | 1.00217953 | 1.000275152 | 1.004087544 |
| WWTR1 | 0.00260248 | 1.01400491 | 1.004867015 | 1.023225906 |
| GREM1 | 0.01345915 | 1.00291715 | 1.000602967 | 1.005236677 |
| NOX4 | 0.02034163 | 1.04692582 | 1.007141771 | 1.088281407 |
| TUBB | 0.00420764 | 1.00158618 | 1.000499716 | 1.002673816 |
