# Supplementary material for: Systematic Analysis of an Invasion-Related 3-Gene Signature and Its Validation as a Prognostic Model for Pancreatic Cancer
Source: Front Oncol. 2021 Dec 15;11:759586. doi: 10.3389/fonc.2021.759586 (PMC8715959; doi:10.3389/fonc.2021.759586)
Supplement: Supplementary file 5 [file Table_3.docx]

Supplement Table 3. 538 differentially expressed genes

| **Genes** | **logFC** | **AveExpr** | **t** | **P.Value** | **adj.P.Val** | **B** |
| --- | --- | --- | --- | --- | --- | --- |
| ADAMTS12 | 2.04807543 | 3.75816854 | 15.0268883 | 1.83E-33 | 2.99E-29 | 65.3656704 |
| PXDN | 1.7319817 | 4.75065976 | 14.8052265 | 8.01E-33 | 6.53E-29 | 63.9148591 |
| ANTXR1 | 2.1362913 | 6.34956229 | 14.3286513 | 1.92E-31 | 8.74E-28 | 60.7869002 |
| COL5A2 | 2.2564889 | 7.38962873 | 14.3121817 | 2.14E-31 | 8.74E-28 | 60.6786179 |
| ADAM12 | 2.24836769 | 3.38204506 | 14.1948151 | 4.69E-31 | 1.51E-27 | 59.9066596 |
| NID2 | 1.66181724 | 3.42908375 | 14.1614961 | 5.86E-31 | 1.51E-27 | 59.6874143 |
| LOX | 2.01565622 | 4.83063733 | 14.1430509 | 6.63E-31 | 1.51E-27 | 59.5660237 |
| ITGA11 | 2.15083499 | 4.00116768 | 14.1270682 | 7.38E-31 | 1.51E-27 | 59.46083 |
| CCN4 | 2.01159032 | 4.03509936 | 14.0922191 | 9.32E-31 | 1.69E-27 | 59.2314313 |
| COL12A1 | 2.39561882 | 6.09938879 | 14.0222058 | 1.49E-30 | 2.43E-27 | 58.7704364 |
| FAP | 1.93637056 | 4.08609294 | 14.0077508 | 1.64E-30 | 2.43E-27 | 58.6752391 |
| COL8A1 | 2.34990843 | 5.07092922 | 13.8008356 | 6.54E-30 | 8.90E-27 | 57.3118784 |
| INHBA | 2.23386559 | 5.04616957 | 13.4375575 | 7.45E-29 | 9.35E-26 | 54.9159024 |
| FKBP7 | 1.16749393 | 3.95664321 | 13.4219191 | 8.27E-29 | 9.64E-26 | 54.8127136 |
| THBS2 | 2.3821015 | 7.60900486 | 13.3495696 | 1.34E-28 | 1.37E-25 | 54.3352883 |
| FN1 | 2.51105776 | 8.76035442 | 13.3493117 | 1.35E-28 | 1.37E-25 | 54.3335863 |
| ANOS1 | 1.43022813 | 2.26120977 | 13.326699 | 1.57E-28 | 1.50E-25 | 54.1843578 |
| CDH11 | 1.94921713 | 4.72666189 | 13.3183252 | 1.66E-28 | 1.50E-25 | 54.1290958 |
| EDNRA | 1.73639449 | 4.64039236 | 13.2506065 | 2.61E-28 | 2.24E-25 | 53.6821744 |
| COL11A1 | 3.15449068 | 4.93505776 | 13.0759301 | 8.40E-28 | 6.85E-25 | 52.5292988 |
| POGLUT2 | 1.17039325 | 3.64413791 | 13.0626981 | 9.18E-28 | 7.13E-25 | 52.4419667 |
| ZNF469 | 1.43174738 | 2.57485257 | 13.0214226 | 1.21E-27 | 8.98E-25 | 52.16955 |
| SPOCK1 | 1.90782108 | 4.2550108 | 13.0105602 | 1.30E-27 | 9.24E-25 | 52.0978594 |
| VCAN | 2.18848682 | 6.29812767 | 13.0008237 | 1.39E-27 | 9.45E-25 | 52.0336005 |
| ZFPM2 | 1.17371901 | 1.95312949 | 12.9767461 | 1.63E-27 | 1.07E-24 | 51.8746949 |
| PRRX1 | 1.88518532 | 4.23629086 | 12.9340047 | 2.17E-27 | 1.36E-24 | 51.5926227 |
| FBN1 | 2.01429107 | 5.47151462 | 12.918437 | 2.41E-27 | 1.46E-24 | 51.4898873 |
| KCND2 | 1.29493207 | 1.8854088 | 12.7752249 | 6.30E-27 | 3.67E-24 | 50.544908 |
| GXYLT2 | 1.57611535 | 2.99522881 | 12.7237809 | 8.89E-27 | 5.00E-24 | 50.2055231 |
| SULF1 | 2.29244655 | 6.71423616 | 12.6963896 | 1.07E-26 | 5.81E-24 | 50.0248359 |
| SPARC | 1.8037173 | 10.4974307 | 12.6904263 | 1.11E-26 | 5.85E-24 | 49.9855008 |
| COL6A3 | 2.21701434 | 7.35413114 | 12.6642858 | 1.32E-26 | 6.75E-24 | 49.8130805 |
| CALU | 1.27355731 | 7.13101332 | 12.652324 | 1.43E-26 | 7.09E-24 | 49.7341855 |
| RAB31 | 1.71041452 | 6.17054897 | 12.6263391 | 1.71E-26 | 8.19E-24 | 49.5628106 |
| COL1A2 | 2.23350178 | 9.99004242 | 12.5831302 | 2.28E-26 | 1.06E-23 | 49.277872 |
| TNFSF4 | 1.41372041 | 2.67386398 | 12.5617723 | 2.63E-26 | 1.19E-23 | 49.1370446 |
| NTM | 1.69865821 | 3.95239189 | 12.5359069 | 3.13E-26 | 1.36E-23 | 48.9665104 |
| RUNX2 | 1.35097949 | 3.06285965 | 12.5337913 | 3.17E-26 | 1.36E-23 | 48.9525626 |
| ST6GAL2 | 1.17381578 | 1.48317911 | 12.5124872 | 3.66E-26 | 1.53E-23 | 48.8121153 |
| GPR176 | 1.18149148 | 3.21217124 | 12.4733108 | 4.75E-26 | 1.94E-23 | 48.5538762 |
| TIMP2 | 1.33947049 | 8.26621013 | 12.4511183 | 5.51E-26 | 2.19E-23 | 48.4076086 |
| HMCN1 | 1.4990838 | 2.40349382 | 12.4433754 | 5.80E-26 | 2.26E-23 | 48.35658 |
| COL5A1 | 2.10098461 | 7.39443372 | 12.4087556 | 7.32E-26 | 2.78E-23 | 48.1284427 |
| TMEM200B | 1.27736121 | 2.80366057 | 12.3116474 | 1.40E-25 | 5.18E-23 | 47.4887232 |
| PTK7 | 1.35306632 | 5.40090249 | 12.3087823 | 1.43E-25 | 5.18E-23 | 47.4698537 |
| CLIC4 | 1.40178509 | 6.76882875 | 12.3044256 | 1.47E-25 | 5.21E-23 | 47.4411607 |
| ADAMTS2 | 1.57723095 | 5.37934659 | 12.2957312 | 1.56E-25 | 5.41E-23 | 47.3839024 |
| LOXL2 | 1.84405202 | 5.31909969 | 12.2374105 | 2.30E-25 | 7.82E-23 | 46.9998958 |
| ZFHX4 | 1.00620072 | 1.38574125 | 12.2170089 | 2.64E-25 | 8.78E-23 | 46.8655936 |
| SH3PXD2B | 1.42490812 | 5.0243744 | 12.1950215 | 3.05E-25 | 9.96E-23 | 46.7208714 |
| MICAL2 | 1.27820919 | 4.73572007 | 12.1865136 | 3.23E-25 | 1.03E-22 | 46.6648776 |
| FNDC1 | 2.26965406 | 4.84243514 | 12.1557166 | 3.97E-25 | 1.25E-22 | 46.4622142 |
| NREP | 1.20682897 | 4.35873605 | 12.1001016 | 5.75E-25 | 1.74E-22 | 46.0963371 |
| NOX4 | 1.39146681 | 2.57997369 | 12.0869354 | 6.28E-25 | 1.86E-22 | 46.0097404 |
| CHSY3 | 1.20751418 | 2.25335033 | 12.0667569 | 7.19E-25 | 2.10E-22 | 45.8770381 |
| LOXL3 | 1.0424119 | 2.91466155 | 12.0567341 | 7.69E-25 | 2.20E-22 | 45.8111308 |
| GLI3 | 1.26487315 | 2.32425221 | 12.0393869 | 8.63E-25 | 2.43E-22 | 45.6970714 |
| RNF144A | 1.29649699 | 4.06039948 | 12.0332332 | 8.99E-25 | 2.46E-22 | 45.6566141 |
| KANK4 | 1.56376681 | 2.02899022 | 12.032203 | 9.05E-25 | 2.46E-22 | 45.6498412 |
| COL3A1 | 2.29577341 | 10.7983947 | 12.0215318 | 9.72E-25 | 2.60E-22 | 45.5796881 |
| P4HA3 | 1.51084036 | 2.84682739 | 12.0175248 | 9.99E-25 | 2.63E-22 | 45.5533474 |
| PLXDC2 | 1.60008162 | 4.75417664 | 12.0051305 | 1.08E-24 | 2.81E-22 | 45.4718767 |
| ARMH4 | 1.01221366 | 1.88683243 | 12.0022333 | 1.11E-24 | 2.82E-22 | 45.4528336 |
| RAI14 | 1.16746685 | 4.79540039 | 11.9896461 | 1.20E-24 | 3.02E-22 | 45.370104 |
| KIRREL1 | 1.43114744 | 4.58003357 | 11.9535296 | 1.53E-24 | 3.78E-22 | 45.1327742 |
| TMEM200A | 1.40020789 | 3.34151944 | 11.9356131 | 1.72E-24 | 4.20E-22 | 45.0150658 |
| CTHRC1 | 2.10593025 | 7.26193486 | 11.8578517 | 2.90E-24 | 6.95E-22 | 44.5043901 |
| PLPP4 | 2.09404275 | 3.94898823 | 11.8254271 | 3.59E-24 | 8.50E-22 | 44.2915528 |
| HSD17B6 | 1.35805416 | 2.37022645 | 11.8137732 | 3.88E-24 | 9.06E-22 | 44.2150709 |
| FKBP14 | 1.03524757 | 3.73088782 | 11.7779373 | 4.93E-24 | 1.10E-21 | 43.9799396 |
| COL4A1 | 1.4926388 | 7.85984457 | 11.7487412 | 5.99E-24 | 1.32E-21 | 43.7884327 |
| TIMP3 | 1.55986062 | 3.02396859 | 11.7440009 | 6.18E-24 | 1.34E-21 | 43.7573443 |
| KIF26B | 1.37506383 | 3.28274571 | 11.715714 | 7.46E-24 | 1.60E-21 | 43.5718615 |
| TNFAIP6 | 1.80677396 | 3.94702939 | 11.7064395 | 7.94E-24 | 1.68E-21 | 43.5110581 |
| ISM1 | 1.89899591 | 4.14114799 | 11.6897267 | 8.87E-24 | 1.86E-21 | 43.4015035 |
| COL8A2 | 1.88585123 | 4.5910123 | 11.681285 | 9.38E-24 | 1.91E-21 | 43.3461742 |
| NUAK1 | 1.3230202 | 3.92533792 | 11.671693 | 1.00E-23 | 2.01E-21 | 43.2833114 |
| FRMD6 | 1.51385815 | 4.0761045 | 11.6542388 | 1.12E-23 | 2.24E-21 | 43.1689378 |
| PLOD2 | 1.51064381 | 4.83205327 | 11.6335485 | 1.29E-23 | 2.53E-21 | 43.0333855 |
| UNC5B | 1.25868853 | 5.19240048 | 11.5058683 | 3.01E-23 | 5.78E-21 | 42.1975689 |
| DDR2 | 1.55515007 | 3.87464246 | 11.4610381 | 4.05E-23 | 7.69E-21 | 41.9043929 |
| GASK1B | 1.35669776 | 4.82168567 | 11.4502363 | 4.36E-23 | 8.17E-21 | 41.8337755 |
| FSTL1 | 1.58162844 | 7.1636417 | 11.4447872 | 4.52E-23 | 8.37E-21 | 41.7981552 |
| SRPX2 | 1.69426462 | 5.05341335 | 11.4229327 | 5.22E-23 | 9.57E-21 | 41.655319 |
| DSE | 1.20177958 | 2.9127304 | 11.3901498 | 6.49E-23 | 1.18E-20 | 41.4411301 |
| VSTM4 | 1.19187319 | 3.18133866 | 11.3825453 | 6.82E-23 | 1.22E-20 | 41.3914583 |
| BNC2 | 1.21564551 | 1.93870152 | 11.37188 | 7.32E-23 | 1.29E-20 | 41.3218015 |
| LAMA4 | 1.32282892 | 4.74264021 | 11.3686849 | 7.48E-23 | 1.29E-20 | 41.300936 |
| VGLL3 | 1.46035162 | 2.6431574 | 11.368168 | 7.51E-23 | 1.29E-20 | 41.2975602 |
| CALHM5 | 1.0065056 | 1.89058627 | 11.3676547 | 7.53E-23 | 1.29E-20 | 41.2942081 |
| NID1 | 1.39846304 | 5.87418899 | 11.3204677 | 1.03E-22 | 1.73E-20 | 40.9861557 |
| PDGFRB | 1.45756266 | 6.78690787 | 11.300341 | 1.18E-22 | 1.96E-20 | 40.8548209 |
| GLT8D2 | 1.41038236 | 4.22110834 | 11.2895159 | 1.26E-22 | 2.07E-20 | 40.7841973 |
| DIO2 | 1.47117945 | 3.25409979 | 11.2892493 | 1.27E-22 | 2.07E-20 | 40.7824579 |
| CTSK | 1.837107 | 7.70840324 | 11.263677 | 1.50E-22 | 2.40E-20 | 40.6156645 |
| SEPTIN11 | 1.13013568 | 5.49409802 | 11.2602661 | 1.53E-22 | 2.43E-20 | 40.5934216 |
| SH3PXD2A | 1.10369327 | 5.42667143 | 11.2273693 | 1.91E-22 | 2.99E-20 | 40.3789523 |
| HTRA1 | 1.41636343 | 8.01654786 | 11.22553 | 1.93E-22 | 3.00E-20 | 40.3669637 |
| CSGALNACT2 | 1.13453441 | 4.91497951 | 11.2070925 | 2.18E-22 | 3.36E-20 | 40.2468081 |
| ZNF521 | 1.30687841 | 2.93254634 | 11.2034591 | 2.23E-22 | 3.41E-20 | 40.2231334 |
| COL10A1 | 2.76157636 | 6.42530711 | 11.199408 | 2.29E-22 | 3.47E-20 | 40.1967377 |
| CHSY1 | 1.1326199 | 4.95986845 | 11.1677623 | 2.83E-22 | 4.20E-20 | 39.9906022 |
| ZNF532 | 1.12375805 | 3.76841509 | 11.1559481 | 3.06E-22 | 4.50E-20 | 39.9136709 |
| GAS1 | 2.02092507 | 4.35101734 | 11.144671 | 3.30E-22 | 4.80E-20 | 39.8402491 |
| AXL | 1.47414475 | 5.43097991 | 11.1323734 | 3.57E-22 | 5.16E-20 | 39.7601974 |
| CERCAM | 1.325754 | 5.53508028 | 11.0906603 | 4.71E-22 | 6.68E-20 | 39.4887762 |
| MRC2 | 1.60805834 | 6.05019527 | 11.060962 | 5.73E-22 | 8.06E-20 | 39.2956392 |
| CALD1 | 1.57412016 | 6.81219848 | 11.0491421 | 6.19E-22 | 8.49E-20 | 39.2187957 |
| LRP1 | 1.28613803 | 6.38009232 | 11.0468592 | 6.29E-22 | 8.55E-20 | 39.2039558 |
| LEF1 | 1.3154712 | 3.71780939 | 11.0318453 | 6.94E-22 | 9.36E-20 | 39.1063721 |
| CORIN | 1.18406657 | 1.70435269 | 11.026362 | 7.20E-22 | 9.63E-20 | 39.0707388 |
| RAB23 | 1.26091667 | 2.73450734 | 11.0247441 | 7.28E-22 | 9.65E-20 | 39.0602255 |
| AEBP1 | 1.8202162 | 8.64657503 | 11.023088 | 7.36E-22 | 9.68E-20 | 39.049464 |
| LUM | 1.92208681 | 9.66045565 | 10.9922535 | 9.01E-22 | 1.18E-19 | 38.8491553 |
| PDPN | 1.72411734 | 5.00395283 | 10.983037 | 9.58E-22 | 1.24E-19 | 38.7893023 |
| ADAMTS7 | 1.1520346 | 2.63858602 | 10.9788762 | 9.85E-22 | 1.27E-19 | 38.7622841 |
| COL4A2 | 1.31859427 | 8.03874522 | 10.9614351 | 1.10E-21 | 1.41E-19 | 38.6490524 |
| LTBP1 | 1.56996911 | 5.70196662 | 10.9507754 | 1.18E-21 | 1.50E-19 | 38.5798625 |
| TWSG1 | 1.07844777 | 5.14144222 | 10.9312601 | 1.35E-21 | 1.69E-19 | 38.4532257 |
| LTBP2 | 1.49993398 | 5.92435079 | 10.919175 | 1.46E-21 | 1.82E-19 | 38.3748251 |
| TENM3 | 1.28406681 | 2.68137224 | 10.911102 | 1.54E-21 | 1.90E-19 | 38.3224618 |
| MMP2 | 2.07350038 | 8.32590368 | 10.8988027 | 1.67E-21 | 2.05E-19 | 38.2426992 |
| SSPN | 1.23073265 | 3.88090938 | 10.8961073 | 1.70E-21 | 2.07E-19 | 38.2252214 |
| COL1A1 | 2.18528726 | 10.7208762 | 10.8894071 | 1.77E-21 | 2.15E-19 | 38.1817792 |
| DCHS1 | 1.22660186 | 3.27216702 | 10.8765924 | 1.93E-21 | 2.32E-19 | 38.098706 |
| PRSS23 | 1.23955641 | 5.39825613 | 10.8738458 | 1.97E-21 | 2.34E-19 | 38.0809032 |
| LAMB1 | 1.11187541 | 6.94860698 | 10.8717442 | 1.99E-21 | 2.36E-19 | 38.0672817 |
| EFEMP2 | 1.2656228 | 5.47705274 | 10.8505253 | 2.29E-21 | 2.67E-19 | 37.9297799 |
| PRR16 | 1.01304243 | 2.48624514 | 10.8240277 | 2.73E-21 | 3.16E-19 | 37.7581437 |
| EFS | 1.10580882 | 3.30828482 | 10.8209423 | 2.78E-21 | 3.20E-19 | 37.7381637 |
| HIP1 | 1.01794261 | 4.16869695 | 10.7907738 | 3.40E-21 | 3.88E-19 | 37.5428606 |
| WNT2 | 1.77525547 | 3.35736838 | 10.7720739 | 3.84E-21 | 4.35E-19 | 37.4218559 |
| ECM2 | 1.3310566 | 3.34533212 | 10.763688 | 4.06E-21 | 4.57E-19 | 37.3676057 |
| LRRC15 | 2.20324278 | 3.33766085 | 10.750623 | 4.42E-21 | 4.91E-19 | 37.2831019 |
| RASGRF2 | 1.06342332 | 2.389978 | 10.7418534 | 4.68E-21 | 5.16E-19 | 37.2263922 |
| CLMP | 1.73369048 | 3.67874528 | 10.7402604 | 4.73E-21 | 5.18E-19 | 37.216092 |
| MXRA5 | 1.97538771 | 6.19105265 | 10.72732 | 5.15E-21 | 5.60E-19 | 37.1324313 |
| ITGAV | 1.23864269 | 6.26558215 | 10.7237093 | 5.27E-21 | 5.66E-19 | 37.1090916 |
| PAPPA | 1.05725332 | 1.67303688 | 10.7061378 | 5.92E-21 | 6.31E-19 | 36.9955305 |
| FZD1 | 1.10621049 | 4.3721652 | 10.6927371 | 6.46E-21 | 6.85E-19 | 36.908951 |
| SYDE1 | 1.02784045 | 4.17195287 | 10.6404963 | 9.10E-21 | 9.59E-19 | 36.5716463 |
| SUGCT | 1.5604742 | 3.2706251 | 10.5851684 | 1.31E-20 | 1.36E-18 | 36.2147918 |
| SYNC | 1.14099721 | 1.65319514 | 10.5817813 | 1.34E-20 | 1.38E-18 | 36.1929584 |
| LATS2 | 1.07780585 | 4.29983631 | 10.5704479 | 1.44E-20 | 1.48E-18 | 36.1199145 |
| CYTH3 | 1.04663539 | 4.65237934 | 10.4795468 | 2.61E-20 | 2.58E-18 | 35.5346828 |
| WNT5A | 1.47209477 | 3.68826966 | 10.4709147 | 2.76E-20 | 2.71E-18 | 35.4791676 |
| ANGPTL2 | 1.48830615 | 5.61672238 | 10.4530829 | 3.10E-20 | 3.03E-18 | 35.3645187 |
| TRAM2 | 1.05409361 | 4.72422216 | 10.4360584 | 3.47E-20 | 3.35E-18 | 35.2551027 |
| LIMS1 | 1.03059043 | 4.9640202 | 10.4224652 | 3.79E-20 | 3.64E-18 | 35.1677683 |
| ITGB5 | 1.14618917 | 7.14424245 | 10.4193387 | 3.87E-20 | 3.69E-18 | 35.147685 |
| MYH9 | 1.0157027 | 8.87446966 | 10.4124168 | 4.04E-20 | 3.84E-18 | 35.1032262 |
| SERPINH1 | 1.28595656 | 7.57908211 | 10.3902419 | 4.67E-20 | 4.38E-18 | 34.9608452 |
| MFAP2 | 1.7490139 | 5.39399889 | 10.3583127 | 5.76E-20 | 5.31E-18 | 34.7559593 |
| DACT1 | 1.26808667 | 4.06273865 | 10.3538845 | 5.92E-20 | 5.40E-18 | 34.7275561 |
| MN1 | 1.2104592 | 3.08324452 | 10.3502063 | 6.07E-20 | 5.50E-18 | 34.7039653 |
| MMP14 | 1.59782275 | 8.69961946 | 10.3459874 | 6.24E-20 | 5.62E-18 | 34.6769095 |
| EDIL3 | 1.53666178 | 4.9470533 | 10.3139683 | 7.68E-20 | 6.81E-18 | 34.4716554 |
| FNDC3B | 1.07806852 | 4.76993182 | 10.2970565 | 8.58E-20 | 7.52E-18 | 34.3633067 |
| TCF4 | 1.10888316 | 3.24877159 | 10.2805727 | 9.55E-20 | 8.33E-18 | 34.2577419 |
| SEC23A | 1.03632806 | 4.45584114 | 10.2595538 | 1.09E-19 | 9.50E-18 | 34.1231933 |
| MSRB3 | 1.45986499 | 4.52229504 | 10.2560479 | 1.12E-19 | 9.67E-18 | 34.100758 |
| APBA2 | 1.02341799 | 2.27410617 | 10.248695 | 1.17E-19 | 1.01E-17 | 34.0537099 |
| LMCD1 | 1.10465767 | 4.21064962 | 10.2443195 | 1.21E-19 | 1.03E-17 | 34.0257168 |
| BMP8A | 1.03470028 | 2.28024136 | 10.243586 | 1.21E-19 | 1.03E-17 | 34.0210244 |
| RFLNB | 1.18638053 | 4.17725281 | 10.2273007 | 1.35E-19 | 1.14E-17 | 33.9168641 |
| PALLD | 1.31365086 | 6.325367 | 10.2078662 | 1.53E-19 | 1.26E-17 | 33.7926164 |
| ARSI | 1.34553447 | 2.62480237 | 10.2077548 | 1.53E-19 | 1.26E-17 | 33.7919038 |
| SMIM3 | 1.17307682 | 5.89842481 | 10.2021165 | 1.59E-19 | 1.30E-17 | 33.7558684 |
| QKI | 1.02720602 | 3.6608647 | 10.1843166 | 1.78E-19 | 1.45E-17 | 33.6421395 |
| POSTN | 2.10414638 | 7.45773101 | 10.1821975 | 1.81E-19 | 1.46E-17 | 33.6286033 |
| ITGA5 | 1.4106399 | 5.92342921 | 10.1748094 | 1.90E-19 | 1.53E-17 | 33.5814152 |
| MYH10 | 1.19776928 | 4.56898402 | 10.1722689 | 1.93E-19 | 1.54E-17 | 33.5651913 |
| EVC | 1.31293157 | 3.36917013 | 10.1490704 | 2.24E-19 | 1.79E-17 | 33.417089 |
| PCDH7 | 1.52529446 | 3.42674927 | 10.1472706 | 2.27E-19 | 1.80E-17 | 33.4056023 |
| COL5A3 | 1.31807758 | 3.89808818 | 10.1454257 | 2.30E-19 | 1.81E-17 | 33.3938283 |
| THY1 | 1.39655534 | 6.78388748 | 10.1370711 | 2.42E-19 | 1.89E-17 | 33.3405179 |
| BASP1 | 1.42595136 | 5.54892479 | 10.1285655 | 2.56E-19 | 1.99E-17 | 33.2862554 |
| C1QTNF6 | 1.16833901 | 3.98927107 | 10.1144718 | 2.81E-19 | 2.17E-17 | 33.196369 |
| OSBPL8 | 1.01098376 | 4.30788769 | 10.1055746 | 2.97E-19 | 2.28E-17 | 33.1396415 |
| ARL4C | 1.47469332 | 6.22400939 | 10.0868415 | 3.36E-19 | 2.55E-17 | 33.0202433 |
| C1QTNF3 | 1.67462787 | 4.07344412 | 10.0679648 | 3.79E-19 | 2.86E-17 | 32.8999897 |
| FIBIN | 1.76131311 | 4.50918963 | 10.0567859 | 4.08E-19 | 3.05E-17 | 32.8288024 |
| THBS1 | 1.98835927 | 7.28818443 | 10.0556626 | 4.11E-19 | 3.06E-17 | 32.8216504 |
| CD109 | 1.47813585 | 3.57010158 | 10.0437162 | 4.44E-19 | 3.29E-17 | 32.7456015 |
| SLC6A6 | 1.26061266 | 5.43896113 | 10.0375673 | 4.62E-19 | 3.41E-17 | 32.7064683 |
| SPSB1 | 1.11567925 | 5.79252531 | 10.025911 | 4.98E-19 | 3.66E-17 | 32.6323012 |
| CNN3 | 1.08834306 | 7.25848968 | 10.0240488 | 5.04E-19 | 3.68E-17 | 32.6204543 |
| GJA1 | 1.27673788 | 6.42649058 | 10.0163444 | 5.29E-19 | 3.84E-17 | 32.5714483 |
| TRPS1 | 1.05388289 | 2.52465716 | 10.0049354 | 5.70E-19 | 4.12E-17 | 32.498896 |
| ABCA1 | 1.13630981 | 3.3989989 | 10.003783 | 5.74E-19 | 4.13E-17 | 32.4915685 |
| CHST11 | 1.30296131 | 4.5309464 | 10.0031031 | 5.77E-19 | 4.13E-17 | 32.4872459 |
| MFAP5 | 2.02495714 | 4.65462432 | 10.0010712 | 5.84E-19 | 4.16E-17 | 32.4743278 |
| CAVIN1 | 1.15212879 | 7.89582421 | 9.98086491 | 6.66E-19 | 4.72E-17 | 32.3459 |
| OLFML1 | 1.2731513 | 3.71101169 | 9.97704584 | 6.82E-19 | 4.80E-17 | 32.3216347 |
| FAM180A | 1.18805413 | 2.26023784 | 9.94971551 | 8.14E-19 | 5.63E-17 | 32.1480592 |
| STON1 | 1.03897863 | 2.55531196 | 9.94117561 | 8.60E-19 | 5.87E-17 | 32.093849 |
| LAMC1 | 1.03579592 | 6.45925274 | 9.92212273 | 9.73E-19 | 6.59E-17 | 31.9729501 |
| SNAI2 | 1.66414504 | 4.8164078 | 9.91855626 | 9.95E-19 | 6.68E-17 | 31.9503265 |
| GPX8 | 1.22555347 | 5.10736106 | 9.90333689 | 1.10E-18 | 7.34E-17 | 31.853809 |
| TSHZ3 | 1.06806555 | 3.16267128 | 9.87907784 | 1.28E-18 | 8.51E-17 | 31.7000501 |
| ACTA2 | 1.55916076 | 8.61784545 | 9.87354327 | 1.33E-18 | 8.78E-17 | 31.6649857 |
| EPYC | 2.40520907 | 2.28230942 | 9.86980002 | 1.36E-18 | 8.93E-17 | 31.6412734 |
| PDGFRA | 1.46429644 | 4.51410256 | 9.85516961 | 1.50E-18 | 9.69E-17 | 31.548619 |
| BMP1 | 1.08184593 | 4.84857847 | 9.80153885 | 2.11E-18 | 1.34E-16 | 31.2093116 |
| ITGB1 | 1.32577537 | 7.84221364 | 9.8014509 | 2.11E-18 | 1.34E-16 | 31.2087556 |
| TWIST1 | 1.6160762 | 3.34614183 | 9.80100276 | 2.12E-18 | 1.34E-16 | 31.2059225 |
| PRKG1 | 1.02623161 | 2.93815281 | 9.78700752 | 2.32E-18 | 1.46E-16 | 31.1174678 |
| CD248 | 1.4291543 | 6.53109511 | 9.78162233 | 2.40E-18 | 1.50E-16 | 31.0834413 |
| DKK3 | 1.19779086 | 6.17427786 | 9.77242772 | 2.55E-18 | 1.57E-16 | 31.0253575 |
| MXRA8 | 1.44082158 | 7.02486939 | 9.76198531 | 2.72E-18 | 1.68E-16 | 30.9594106 |
| MFGE8 | 1.1862937 | 6.10009165 | 9.74149717 | 3.11E-18 | 1.89E-16 | 30.8300821 |
| MAF | 1.0537469 | 4.33317786 | 9.73680032 | 3.20E-18 | 1.94E-16 | 30.8004452 |
| NOTCH3 | 1.1457549 | 5.72077099 | 9.72419675 | 3.47E-18 | 2.10E-16 | 30.7209381 |
| FBLN2 | 1.60054354 | 5.99176924 | 9.71582805 | 3.66E-18 | 2.19E-16 | 30.6681628 |
| ROR2 | 1.210081 | 3.44060538 | 9.70086574 | 4.03E-18 | 2.39E-16 | 30.5738401 |
| FBXL7 | 1.09106183 | 3.25218851 | 9.68602247 | 4.43E-18 | 2.60E-16 | 30.4803108 |
| PMP22 | 1.27125331 | 6.5730296 | 9.67740832 | 4.68E-18 | 2.72E-16 | 30.4260517 |
| LOXL1 | 1.22754133 | 5.37919515 | 9.67716578 | 4.69E-18 | 2.72E-16 | 30.4245242 |
| MARVELD1 | 1.02722665 | 6.29147744 | 9.67608354 | 4.72E-18 | 2.73E-16 | 30.4177084 |
| BGN | 1.38062261 | 10.2540082 | 9.6680181 | 4.97E-18 | 2.87E-16 | 30.3669208 |
| GFPT2 | 1.58240762 | 4.30210016 | 9.6639877 | 5.10E-18 | 2.93E-16 | 30.3415465 |
| CMTM3 | 1.34437977 | 6.0678421 | 9.65589873 | 5.38E-18 | 3.08E-16 | 30.2906301 |
| GLI2 | 1.03209962 | 2.57197745 | 9.65329427 | 5.47E-18 | 3.11E-16 | 30.2742389 |
| GREM1 | 2.12893971 | 4.73612197 | 9.64944025 | 5.60E-18 | 3.17E-16 | 30.2499861 |
| LRRC17 | 1.33926819 | 3.30561301 | 9.62389295 | 6.60E-18 | 3.70E-16 | 30.0892953 |
| GNB4 | 1.15722164 | 3.60493973 | 9.62089815 | 6.72E-18 | 3.76E-16 | 30.0704668 |
| ITPRIP | 1.07990041 | 4.46086811 | 9.62041132 | 6.75E-18 | 3.76E-16 | 30.0674063 |
| HEG1 | 1.20225061 | 4.71436187 | 9.61624655 | 6.93E-18 | 3.83E-16 | 30.0412254 |
| FERMT2 | 1.1700196 | 4.1756551 | 9.60368616 | 7.51E-18 | 4.11E-16 | 29.9622886 |
| ITGA1 | 1.01187229 | 4.21483731 | 9.58556133 | 8.43E-18 | 4.59E-16 | 29.8484377 |
| GPNMB | 1.6658273 | 6.47000798 | 9.58497304 | 8.46E-18 | 4.59E-16 | 29.8447434 |
| SCUBE2 | 1.30576441 | 3.08649069 | 9.57150812 | 9.22E-18 | 4.96E-16 | 29.7602086 |
| GPR68 | 1.26534909 | 3.64756433 | 9.54817705 | 1.07E-17 | 5.70E-16 | 29.6138202 |
| RUNX1 | 1.0913074 | 4.76433313 | 9.54250858 | 1.11E-17 | 5.86E-16 | 29.5782709 |
| SSC5D | 1.30611494 | 3.82821762 | 9.53307568 | 1.18E-17 | 6.20E-16 | 29.519128 |
| FBXO32 | 1.25063336 | 5.21258211 | 9.5303385 | 1.20E-17 | 6.29E-16 | 29.5019697 |
| PDGFC | 1.42080677 | 4.83870049 | 9.52456103 | 1.24E-17 | 6.50E-16 | 29.4657582 |
| RECK | 1.06256542 | 2.97094386 | 9.52135333 | 1.27E-17 | 6.62E-16 | 29.4456563 |
| RECQL | 1.09684018 | 4.11211822 | 9.51239788 | 1.34E-17 | 6.95E-16 | 29.3895458 |
| DPYSL3 | 1.37885849 | 6.45580115 | 9.50750586 | 1.39E-17 | 7.11E-16 | 29.3589019 |
| LRRC32 | 1.15995948 | 6.22096912 | 9.49660891 | 1.49E-17 | 7.58E-16 | 29.2906607 |
| ZEB2 | 1.06313925 | 2.75247438 | 9.48835583 | 1.57E-17 | 7.96E-16 | 29.2389931 |
| XYLT1 | 1.07315636 | 3.47359618 | 9.48248385 | 1.63E-17 | 8.24E-16 | 29.202241 |
| NKX3-2 | 1.10029954 | 1.99517962 | 9.46681632 | 1.80E-17 | 9.07E-16 | 29.104215 |
| ISLR | 1.58813842 | 7.87297595 | 9.41723465 | 2.46E-17 | 1.23E-15 | 28.7943457 |
| IL1R1 | 1.38370124 | 5.833249 | 9.40444793 | 2.67E-17 | 1.32E-15 | 28.7145187 |
| ZEB1 | 1.13638383 | 4.13564527 | 9.3654415 | 3.42E-17 | 1.65E-15 | 28.4712235 |
| ITGBL1 | 1.72726055 | 4.00789657 | 9.349748 | 3.78E-17 | 1.81E-15 | 28.3734327 |
| SORCS2 | 1.25106138 | 2.67400369 | 9.33740932 | 4.08E-17 | 1.93E-15 | 28.296585 |
| SCARF2 | 1.2759508 | 4.37365595 | 9.33428591 | 4.16E-17 | 1.96E-15 | 28.2771372 |
| RASSF8 | 1.09920927 | 3.27931654 | 9.32669354 | 4.37E-17 | 2.04E-15 | 28.2298726 |
| KIAA1217 | 1.05754027 | 5.03296709 | 9.28447742 | 5.71E-17 | 2.62E-15 | 27.9673016 |
| SGCD | 1.23517905 | 2.38961475 | 9.27765857 | 5.96E-17 | 2.72E-15 | 27.9249283 |
| FZD7 | 1.14887144 | 4.12829047 | 9.27571561 | 6.03E-17 | 2.75E-15 | 27.9128564 |
| PODNL1 | 1.30204824 | 3.18800578 | 9.27380539 | 6.10E-17 | 2.76E-15 | 27.9009887 |
| MRVI1 | 1.24992883 | 4.01636303 | 9.273315 | 6.12E-17 | 2.76E-15 | 27.8979422 |
| DAB2 | 1.02810497 | 5.01387322 | 9.26587233 | 6.42E-17 | 2.88E-15 | 27.8517118 |
| ASPN | 1.70586727 | 6.46124482 | 9.24581017 | 7.28E-17 | 3.25E-15 | 27.7271582 |
| ADAM19 | 1.42471353 | 4.06517781 | 9.24363268 | 7.38E-17 | 3.28E-15 | 27.713645 |
| DCN | 1.57777876 | 7.13839669 | 9.23310501 | 7.89E-17 | 3.50E-15 | 27.6483275 |
| SPON1 | 1.72294656 | 5.44374441 | 9.22858206 | 8.12E-17 | 3.58E-15 | 27.6202733 |
| PDGFRL | 1.36828711 | 4.0703646 | 9.22844189 | 8.13E-17 | 3.58E-15 | 27.619404 |
| SEMA3C | 1.61779544 | 5.10309735 | 9.17875541 | 1.11E-16 | 4.86E-15 | 27.3115321 |
| RCN3 | 1.24544969 | 6.43260912 | 9.17528744 | 1.14E-16 | 4.93E-15 | 27.2900652 |
| OLFML2B | 1.55385731 | 5.86256061 | 9.17112649 | 1.17E-16 | 5.05E-15 | 27.2643124 |
| SFRP2 | 2.3180928 | 9.39013735 | 9.16767892 | 1.19E-16 | 5.15E-15 | 27.242978 |
| TMEM45A | 1.28409502 | 4.00731068 | 9.15727431 | 1.27E-16 | 5.48E-15 | 27.1786085 |
| PRDM1 | 1.05603141 | 3.58318502 | 9.15417149 | 1.30E-16 | 5.57E-15 | 27.1594175 |
| COLEC12 | 1.61739246 | 3.284827 | 9.1270035 | 1.54E-16 | 6.54E-15 | 26.9914808 |
| COL6A1 | 1.3974182 | 8.09276893 | 9.12081102 | 1.60E-16 | 6.77E-15 | 26.9532271 |
| PRKD1 | 1.02068972 | 2.8954254 | 9.11983958 | 1.61E-16 | 6.79E-15 | 26.947227 |
| PLAU | 1.79216476 | 7.20755343 | 9.09282874 | 1.91E-16 | 7.90E-15 | 26.7804838 |
| FKBP10 | 1.12469186 | 6.53437561 | 9.09020296 | 1.94E-16 | 8.01E-15 | 26.7642838 |
| F2R | 1.30931995 | 6.12851748 | 9.08513027 | 2.00E-16 | 8.23E-15 | 26.732992 |
| GPR1 | 1.07539567 | 1.33446105 | 9.07695814 | 2.11E-16 | 8.64E-15 | 26.682594 |
| PLXNC1 | 1.13055817 | 3.4606649 | 9.037896 | 2.69E-16 | 1.08E-14 | 26.4419211 |
| EMILIN1 | 1.41237673 | 6.88059088 | 9.02674443 | 2.89E-16 | 1.16E-14 | 26.3732822 |
| LGALS1 | 1.31895937 | 9.65501635 | 9.01396551 | 3.13E-16 | 1.25E-14 | 26.2946646 |
| WT1 | 1.08245703 | 1.39035231 | 9.00724594 | 3.26E-16 | 1.30E-14 | 26.2533413 |
| CRISPLD2 | 1.43046086 | 6.01864739 | 8.99937951 | 3.43E-16 | 1.35E-14 | 26.2049795 |
| MITF | 1.06958714 | 3.17483711 | 8.99702982 | 3.48E-16 | 1.37E-14 | 26.1905369 |
| FCGR2A | 1.35131076 | 4.48337658 | 8.99478817 | 3.53E-16 | 1.38E-14 | 26.1767596 |
| OLFML3 | 1.35322445 | 5.89332939 | 8.98781014 | 3.68E-16 | 1.44E-14 | 26.1338806 |
| COL6A2 | 1.38074765 | 8.6341739 | 8.98667337 | 3.71E-16 | 1.45E-14 | 26.1268964 |
| PTGFRN | 1.03522654 | 5.95312881 | 8.96822704 | 4.16E-16 | 1.62E-14 | 26.0136106 |
| GAS7 | 1.17197878 | 3.99228564 | 8.9632594 | 4.29E-16 | 1.66E-14 | 25.9831171 |
| SMO | 1.01910819 | 3.86377261 | 8.94019214 | 4.96E-16 | 1.89E-14 | 25.8416027 |
| MSR1 | 1.40216284 | 3.67909258 | 8.92589826 | 5.42E-16 | 2.06E-14 | 25.7539799 |
| ROBO1 | 1.06736547 | 3.26502009 | 8.89831253 | 6.44E-16 | 2.42E-14 | 25.5850252 |
| HSPG2 | 1.2963857 | 6.24538072 | 8.88718245 | 6.90E-16 | 2.57E-14 | 25.5169124 |
| CYBRD1 | 1.37471591 | 6.40760661 | 8.82022793 | 1.05E-15 | 3.79E-14 | 25.1078538 |
| GPC6 | 1.26045225 | 3.58061626 | 8.81479015 | 1.08E-15 | 3.92E-14 | 25.0746836 |
| TREM1 | 1.33424097 | 2.66662737 | 8.77590364 | 1.38E-15 | 4.90E-14 | 24.8377082 |
| ALDH1L2 | 1.1024675 | 2.70118423 | 8.75393319 | 1.58E-15 | 5.56E-14 | 24.7039995 |
| PCOLCE | 1.2582512 | 6.4483284 | 8.7517675 | 1.60E-15 | 5.63E-14 | 24.6908266 |
| CDK14 | 1.09686531 | 4.15388853 | 8.73579003 | 1.77E-15 | 6.17E-14 | 24.5936818 |
| SCUBE3 | 1.37711869 | 2.02090675 | 8.73045951 | 1.83E-15 | 6.36E-14 | 24.5612872 |
| MAP1A | 1.02666767 | 3.46003634 | 8.72466682 | 1.89E-15 | 6.57E-14 | 24.5260926 |
| GALNT15 | 1.12504468 | 2.09014115 | 8.66485772 | 2.74E-15 | 9.27E-14 | 24.1632521 |
| WIPF1 | 1.10231478 | 4.55437746 | 8.64773002 | 3.04E-15 | 1.03E-13 | 24.0595275 |
| CILP2 | 1.46100485 | 2.90581936 | 8.62934869 | 3.41E-15 | 1.14E-13 | 23.9483026 |
| KCNE4 | 1.22532035 | 4.30546641 | 8.62717964 | 3.45E-15 | 1.15E-13 | 23.935184 |
| GLIPR1 | 1.12509658 | 3.66804552 | 8.57389576 | 4.79E-15 | 1.56E-13 | 23.6133387 |
| SERPINE1 | 1.71398103 | 7.78561819 | 8.57312328 | 4.81E-15 | 1.57E-13 | 23.6086788 |
| LAMA2 | 1.40685163 | 4.09201125 | 8.57096874 | 4.88E-15 | 1.59E-13 | 23.5956825 |
| NPR3 | 1.39856929 | 2.85911815 | 8.57071197 | 4.89E-15 | 1.59E-13 | 23.5941337 |
| PDLIM3 | 1.29330248 | 4.18174797 | 8.56738306 | 4.99E-15 | 1.61E-13 | 23.5740564 |
| CCDC80 | 1.68011734 | 5.80743646 | 8.5542702 | 5.41E-15 | 1.73E-13 | 23.4950012 |
| EGFL6 | 1.11267802 | 2.253183 | 8.53888099 | 5.94E-15 | 1.88E-13 | 23.4022856 |
| TMEM158 | 1.35679238 | 4.48243717 | 8.53280137 | 6.17E-15 | 1.92E-13 | 23.3656766 |
| PCDH18 | 1.07922089 | 3.04236404 | 8.49687169 | 7.68E-15 | 2.36E-13 | 23.1495427 |
| GLIS2 | 1.06386118 | 5.19913538 | 8.49276252 | 7.88E-15 | 2.42E-13 | 23.1248482 |
| OSMR | 1.02810481 | 5.28746835 | 8.44666419 | 1.04E-14 | 3.15E-13 | 22.8481578 |
| SULF2 | 1.12358259 | 6.57436401 | 8.43425819 | 1.13E-14 | 3.38E-13 | 22.7738027 |
| CPXM1 | 1.56458544 | 5.45621293 | 8.43153031 | 1.15E-14 | 3.42E-13 | 22.7574594 |
| ADGRA2 | 1.14016356 | 4.80388794 | 8.43063306 | 1.15E-14 | 3.43E-13 | 22.7520843 |
| NRP2 | 1.17236233 | 4.32568349 | 8.41449711 | 1.27E-14 | 3.76E-13 | 22.6554605 |
| MDFIC | 1.07369201 | 4.35315915 | 8.40692145 | 1.33E-14 | 3.92E-13 | 22.6101237 |
| HOPX | 1.14776687 | 4.32834523 | 8.38338281 | 1.54E-14 | 4.50E-13 | 22.4693667 |
| LHFPL6 | 1.12063184 | 5.95265163 | 8.37776892 | 1.59E-14 | 4.62E-13 | 22.4358215 |
| SERPINF1 | 1.4377221 | 7.76550712 | 8.36422906 | 1.73E-14 | 4.99E-13 | 22.354955 |
| TGFB2 | 1.37630218 | 3.35371129 | 8.3273995 | 2.16E-14 | 6.17E-13 | 22.1352758 |
| IL1RAP | 1.02489106 | 2.96860188 | 8.32655084 | 2.17E-14 | 6.19E-13 | 22.1302187 |
| CCDC8 | 1.02821644 | 4.18955211 | 8.3026287 | 2.51E-14 | 7.05E-13 | 21.9877599 |
| FLNA | 1.32382437 | 8.16421881 | 8.28138984 | 2.85E-14 | 7.94E-13 | 21.8614298 |
| COPZ2 | 1.01695304 | 4.80102328 | 8.26487856 | 3.15E-14 | 8.64E-13 | 21.7633174 |
| MOXD1 | 1.40134631 | 4.46772805 | 8.2623779 | 3.20E-14 | 8.74E-13 | 21.7484655 |
| VIM | 1.04458803 | 9.00801659 | 8.25637358 | 3.32E-14 | 9.01E-13 | 21.712813 |
| FCGR3A | 1.47916635 | 5.68435889 | 8.24769967 | 3.50E-14 | 9.46E-13 | 21.6613291 |
| EFEMP1 | 1.45764802 | 6.09879978 | 8.22193229 | 4.09E-14 | 1.10E-12 | 21.5085277 |
| DCBLD2 | 1.39221221 | 4.33088365 | 8.21145771 | 4.36E-14 | 1.16E-12 | 21.4464736 |
| COL15A1 | 1.25962141 | 5.91424774 | 8.19321748 | 4.86E-14 | 1.29E-12 | 21.3384977 |
| CCN2 | 1.47486224 | 8.67673205 | 8.19313769 | 4.87E-14 | 1.29E-12 | 21.3380256 |
| MSN | 1.05871686 | 7.6154886 | 8.18675756 | 5.06E-14 | 1.33E-12 | 21.3002828 |
| NEXN | 1.29200834 | 3.73430644 | 8.16657845 | 5.71E-14 | 1.48E-12 | 21.1809963 |
| PPP1R18 | 1.00668376 | 6.06260329 | 8.16432868 | 5.79E-14 | 1.49E-12 | 21.1677052 |
| F13A1 | 1.60194045 | 4.97856856 | 8.15356229 | 6.18E-14 | 1.58E-12 | 21.1041227 |
| MMP19 | 1.25903156 | 3.97991197 | 8.14854447 | 6.37E-14 | 1.62E-12 | 21.0745021 |
| C5orf46 | 1.4368401 | 2.84319352 | 8.13806073 | 6.78E-14 | 1.72E-12 | 21.0126423 |
| ZCCHC24 | 1.11706365 | 4.96828732 | 8.10467981 | 8.29E-14 | 2.07E-12 | 20.8159165 |
| C1S | 1.35303318 | 7.78837059 | 8.07749348 | 9.75E-14 | 2.40E-12 | 20.6559692 |
| RFTN1 | 1.00547792 | 4.91447673 | 8.06352483 | 1.06E-13 | 2.59E-12 | 20.5738818 |
| TAGLN | 1.37982299 | 8.37264123 | 8.02456221 | 1.34E-13 | 3.17E-12 | 20.3452618 |
| LZTS1 | 1.01907414 | 3.60291452 | 8.00823299 | 1.48E-13 | 3.47E-12 | 20.2495992 |
| CRISPLD1 | 1.06672979 | 2.75561878 | 7.99314323 | 1.62E-13 | 3.75E-12 | 20.1612784 |
| AOC3 | 1.25452709 | 4.96070005 | 7.98152346 | 1.73E-13 | 4.00E-12 | 20.0933203 |
| AQP9 | 1.30087268 | 2.55606485 | 7.974683 | 1.80E-13 | 4.12E-12 | 20.0533355 |
| PCDHGC3 | 1.03162329 | 3.37269095 | 7.97016095 | 1.85E-13 | 4.22E-12 | 20.0269114 |
| SLC1A3 | 1.22536277 | 2.94986504 | 7.96598963 | 1.90E-13 | 4.29E-12 | 20.0025429 |
| ETS1 | 1.01442091 | 5.8341027 | 7.93669585 | 2.26E-13 | 5.02E-12 | 19.8315801 |
| MYOF | 1.23015125 | 6.30640915 | 7.93082472 | 2.34E-13 | 5.17E-12 | 19.797351 |
| TGFB3 | 1.35056717 | 5.13012899 | 7.91018783 | 2.65E-13 | 5.77E-12 | 19.6771313 |
| MATN3 | 1.73031553 | 4.66783779 | 7.89481332 | 2.90E-13 | 6.29E-12 | 19.5876639 |
| IGFBP3 | 1.42917419 | 8.15956456 | 7.86709739 | 3.42E-13 | 7.25E-12 | 19.4265886 |
| EMP1 | 1.2994041 | 6.38372807 | 7.85272102 | 3.72E-13 | 7.86E-12 | 19.3431447 |
| C5AR1 | 1.17202271 | 4.52047952 | 7.84857345 | 3.81E-13 | 8.03E-12 | 19.3190847 |
| FPR3 | 1.39600979 | 4.15253972 | 7.84653896 | 3.86E-13 | 8.11E-12 | 19.3072849 |
| MYLK | 1.33500648 | 3.92741156 | 7.84607733 | 3.87E-13 | 8.12E-12 | 19.3046078 |
| IGFBP5 | 1.51434603 | 8.64548807 | 7.83747586 | 4.07E-13 | 8.51E-12 | 19.254738 |
| XIRP1 | 1.08594309 | 1.30312233 | 7.83699731 | 4.08E-13 | 8.52E-12 | 19.2519642 |
| SLC2A3 | 1.29870675 | 4.91176206 | 7.82581947 | 4.36E-13 | 9.06E-12 | 19.1871983 |
| CCN1 | 1.52713197 | 7.78387923 | 7.80578306 | 4.91E-13 | 1.01E-11 | 19.0712164 |
| SLIT2 | 1.14036402 | 3.0122306 | 7.77861006 | 5.76E-13 | 1.17E-11 | 18.914154 |
| CLEC5A | 1.05526884 | 2.4708763 | 7.76944189 | 6.08E-13 | 1.22E-11 | 18.8612213 |
| ADAMTS4 | 1.11480179 | 3.84870091 | 7.76299606 | 6.32E-13 | 1.27E-11 | 18.8240242 |
| ICAM1 | 1.18704123 | 5.54430797 | 7.74860955 | 6.88E-13 | 1.37E-11 | 18.7410582 |
| NOTCH2 | 1.02799606 | 4.54668191 | 7.72322597 | 7.99E-13 | 1.58E-11 | 18.5948569 |
| C1R | 1.17028928 | 7.74047094 | 7.72233215 | 8.03E-13 | 1.59E-11 | 18.5897131 |
| PYGL | 1.04126044 | 4.45512118 | 7.69161569 | 9.61E-13 | 1.87E-11 | 18.4131222 |
| PILRA | 1.03171454 | 3.60127834 | 7.68868474 | 9.78E-13 | 1.90E-11 | 18.3962902 |
| GEM | 1.2481725 | 5.87340523 | 7.65536384 | 1.19E-12 | 2.25E-11 | 18.205157 |
| BCAT1 | 1.1775025 | 3.30548779 | 7.63386268 | 1.35E-12 | 2.53E-11 | 18.0820433 |
| RGS16 | 1.30294331 | 5.15826238 | 7.62817139 | 1.39E-12 | 2.60E-11 | 18.0494845 |
| OLR1 | 1.65764569 | 4.47351234 | 7.61554407 | 1.50E-12 | 2.78E-11 | 17.9772894 |
| PLIN2 | 1.10245873 | 4.92175458 | 7.6093032 | 1.56E-12 | 2.88E-11 | 17.9416303 |
| MMP11 | 2.06919739 | 7.49510282 | 7.60030454 | 1.64E-12 | 3.03E-11 | 17.8902395 |
| FCER1G | 1.18732718 | 6.5531704 | 7.5975121 | 1.67E-12 | 3.07E-11 | 17.8742983 |
| SLC37A2 | 1.05462044 | 2.83344785 | 7.58733192 | 1.77E-12 | 3.23E-11 | 17.8162077 |
| BICC1 | 1.19214955 | 4.52952341 | 7.56794212 | 1.98E-12 | 3.56E-11 | 17.7056738 |
| CLEC11A | 1.00047094 | 5.82094425 | 7.5526383 | 2.16E-12 | 3.85E-11 | 17.618534 |
| FILIP1L | 1.05631794 | 4.94838787 | 7.54936889 | 2.21E-12 | 3.90E-11 | 17.5999296 |
| SNAI1 | 1.03372663 | 4.25566061 | 7.51537305 | 2.69E-12 | 4.68E-11 | 17.4067221 |
| HTRA3 | 1.4181242 | 7.2336623 | 7.50226434 | 2.90E-12 | 5.00E-11 | 17.332341 |
| FPR1 | 1.26020005 | 3.88540377 | 7.49142176 | 3.09E-12 | 5.30E-11 | 17.2708689 |
| PODN | 1.22154977 | 5.32516168 | 7.48967029 | 3.12E-12 | 5.34E-11 | 17.2609432 |
| MYL9 | 1.16739751 | 8.42344271 | 7.48226588 | 3.25E-12 | 5.54E-11 | 17.2189951 |
| GJB2 | 1.71786566 | 6.19634501 | 7.47787818 | 3.34E-12 | 5.67E-11 | 17.1941476 |
| NCF2 | 1.18453433 | 4.7922895 | 7.44169136 | 4.11E-12 | 6.87E-11 | 16.9895102 |
| RAB34 | 1.04875446 | 5.40183087 | 7.40719838 | 5.02E-12 | 8.22E-11 | 16.7949323 |
| SPON2 | 1.12374803 | 6.02000208 | 7.39414956 | 5.41E-12 | 8.80E-11 | 16.7214459 |
| SYNDIG1 | 1.13517585 | 3.24158224 | 7.35081535 | 6.94E-12 | 1.10E-10 | 16.4778921 |
| ALOX5AP | 1.19407721 | 5.94824355 | 7.34285184 | 7.26E-12 | 1.15E-10 | 16.4332165 |
| IGFL2 | 1.67303116 | 3.34304929 | 7.33140246 | 7.76E-12 | 1.22E-10 | 16.3690301 |
| FGF7 | 1.2869493 | 2.60972176 | 7.32503388 | 8.04E-12 | 1.26E-10 | 16.3333501 |
| ANXA1 | 1.31346767 | 7.4356373 | 7.31707131 | 8.42E-12 | 1.31E-10 | 16.288763 |
| SVEP1 | 1.13639407 | 2.94135604 | 7.30456705 | 9.04E-12 | 1.39E-10 | 16.2187965 |
| CLEC7A | 1.03472336 | 2.97914093 | 7.26187067 | 1.15E-11 | 1.74E-10 | 15.9803749 |
| GBP1 | 1.05412456 | 4.41952717 | 7.23774421 | 1.32E-11 | 1.97E-10 | 15.845982 |
| CD86 | 1.04962579 | 3.48499356 | 7.22783619 | 1.40E-11 | 2.08E-10 | 15.7908608 |
| ADAMTS14 | 1.07667519 | 3.14025267 | 7.22391891 | 1.43E-11 | 2.12E-10 | 15.7690791 |
| FMOD | 1.01759082 | 6.58127714 | 7.22273552 | 1.44E-11 | 2.13E-10 | 15.7625002 |
| FMO1 | 1.05658583 | 2.38647918 | 7.22167228 | 1.45E-11 | 2.14E-10 | 15.7565898 |
| AKAP12 | 1.21977262 | 4.96729569 | 7.21082789 | 1.54E-11 | 2.26E-10 | 15.6963339 |
| COL16A1 | 1.05074384 | 5.74472438 | 7.20382065 | 1.61E-11 | 2.34E-10 | 15.657425 |
| HAVCR2 | 1.04677379 | 3.76188483 | 7.19690188 | 1.67E-11 | 2.43E-10 | 15.6190275 |
| LAMP5 | 1.42173988 | 5.09897545 | 7.19528883 | 1.69E-11 | 2.44E-10 | 15.6100784 |
| ALDH1A3 | 1.21183092 | 4.02315398 | 7.16797995 | 1.97E-11 | 2.78E-10 | 15.4587355 |
| SLC11A1 | 1.11674474 | 3.29172553 | 7.1433543 | 2.26E-11 | 3.14E-10 | 15.3225327 |
| COMP | 1.85652502 | 6.70984178 | 7.12103294 | 2.57E-11 | 3.52E-10 | 15.1992974 |
| CD163 | 1.47118248 | 4.82969605 | 7.1128966 | 2.69E-11 | 3.67E-10 | 15.1544299 |
| SAMSN1 | 1.08028791 | 3.26211877 | 7.10483996 | 2.81E-11 | 3.83E-10 | 15.1100298 |
| FBLN1 | 1.39768716 | 6.87736441 | 7.10393962 | 2.82E-11 | 3.84E-10 | 15.1050697 |
| TGFBI | 1.3557088 | 6.55199156 | 7.09887061 | 2.91E-11 | 3.95E-10 | 15.0771507 |
| MME | 1.11020764 | 2.02106889 | 7.02950755 | 4.29E-11 | 5.61E-10 | 14.6962273 |
| EGR2 | 1.17900363 | 4.05951922 | 7.02437146 | 4.42E-11 | 5.76E-10 | 14.6681043 |
| SPHK1 | 1.00345435 | 4.71398513 | 6.92395896 | 7.74E-11 | 9.52E-10 | 14.1206243 |
| ARHGAP23 | 1.01278347 | 4.12810703 | 6.90164178 | 8.76E-11 | 1.06E-09 | 13.9995534 |
| IBSP | 1.12053522 | 1.56981116 | 6.89665509 | 9.00E-11 | 1.09E-09 | 13.9725311 |
| CCR1 | 1.03028575 | 3.27668457 | 6.85748372 | 1.12E-10 | 1.32E-09 | 13.7606569 |
| LAPTM5 | 1.08573215 | 7.57776333 | 6.76205913 | 1.89E-10 | 2.12E-09 | 13.2474461 |
| OMD | 1.36392102 | 3.14740029 | 6.75111037 | 2.01E-10 | 2.23E-09 | 13.1888305 |
| C3AR1 | 1.11872964 | 4.37940662 | 6.701037 | 2.64E-10 | 2.83E-09 | 12.92147 |
| RSAD2 | 1.08450788 | 3.6261237 | 6.68374761 | 2.91E-10 | 3.09E-09 | 12.829429 |
| PRELP | 1.23934466 | 5.82403291 | 6.66078323 | 3.29E-10 | 3.43E-09 | 12.7073957 |
| SLCO2B1 | 1.02350243 | 4.22637268 | 6.65529421 | 3.39E-10 | 3.53E-09 | 12.6782639 |
| ITGB2 | 1.12004805 | 5.62904567 | 6.61060719 | 4.32E-10 | 4.41E-09 | 12.4416328 |
| C1QC | 1.13405891 | 7.92048537 | 6.60282318 | 4.51E-10 | 4.58E-09 | 12.400512 |
| SLAMF8 | 1.02357104 | 4.03045586 | 6.57229691 | 5.32E-10 | 5.30E-09 | 12.2395321 |
| MNDA | 1.12788582 | 3.75536744 | 6.55731597 | 5.77E-10 | 5.68E-09 | 12.1606954 |
| EVI2A | 1.04673083 | 3.70238711 | 6.54187698 | 6.27E-10 | 6.13E-09 | 12.0795625 |
| ELN | 1.13695038 | 6.02648575 | 6.53628808 | 6.46E-10 | 6.29E-09 | 12.0502211 |
| TMEM119 | 1.17051718 | 5.68595756 | 6.52518425 | 6.86E-10 | 6.65E-09 | 11.9919719 |
| CPXM2 | 1.08665914 | 4.42916899 | 6.50018764 | 7.84E-10 | 7.47E-09 | 11.8610643 |
| CDH2 | 1.16604378 | 3.04976943 | 6.4707613 | 9.19E-10 | 8.61E-09 | 11.7073526 |
| VSIG4 | 1.25892141 | 5.14451081 | 6.46699644 | 9.37E-10 | 8.76E-09 | 11.6877174 |
| HSD11B1 | 1.13258858 | 4.02884004 | 6.45406405 | 1.00E-09 | 9.30E-09 | 11.6203234 |
| CALB2 | 1.557655 | 3.87061749 | 6.45316074 | 1.01E-09 | 9.33E-09 | 11.6156191 |
| SRGN | 1.09387597 | 7.73585291 | 6.42027176 | 1.20E-09 | 1.09E-08 | 11.4446165 |
| CXCL8 | 1.50744622 | 5.21128799 | 6.40394529 | 1.31E-09 | 1.17E-08 | 11.3599298 |
| MS4A4A | 1.12579263 | 4.15949671 | 6.39870249 | 1.35E-09 | 1.20E-08 | 11.3327634 |
| SFRP4 | 1.53282326 | 6.79190911 | 6.36023271 | 1.66E-09 | 1.44E-08 | 11.1338504 |
| ST6GALNAC5 | 1.01399492 | 2.39322645 | 6.35692975 | 1.68E-09 | 1.46E-08 | 11.1168069 |
| ALOX15B | 1.18350428 | 2.62725828 | 6.34963104 | 1.75E-09 | 1.51E-08 | 11.0791648 |
| MEGF6 | 1.01719885 | 4.46338016 | 6.34296865 | 1.81E-09 | 1.56E-08 | 11.0448281 |
| HAS2 | 1.04775581 | 2.71302502 | 6.34259316 | 1.82E-09 | 1.57E-08 | 11.0428936 |
| CFH | 1.01100042 | 5.33689606 | 6.33920974 | 1.85E-09 | 1.59E-08 | 11.0254654 |
| PLAT | 1.36319229 | 7.02512532 | 6.29621839 | 2.32E-09 | 1.95E-08 | 10.8045238 |
| NPTX1 | 1.07530139 | 2.56159513 | 6.27829214 | 2.55E-09 | 2.13E-08 | 10.7126774 |
| PLA2G7 | 1.07529556 | 3.60317351 | 6.27794502 | 2.56E-09 | 2.13E-08 | 10.7109006 |
| IL2RA | 1.13653177 | 2.85184388 | 6.27196664 | 2.64E-09 | 2.19E-08 | 10.6803079 |
| PLAUR | 1.06855216 | 6.07526727 | 6.21963587 | 3.47E-09 | 2.79E-08 | 10.4133111 |
| SCT | 1.10226774 | 3.30328619 | 6.16186383 | 4.69E-09 | 3.67E-08 | 10.1202156 |
| MS4A7 | 1.06032199 | 4.07970875 | 6.14940396 | 5.01E-09 | 3.88E-08 | 10.0572335 |
| CAV1 | 1.04424641 | 6.01031183 | 6.13563396 | 5.38E-09 | 4.13E-08 | 9.98772484 |
| NDNF | 1.05637461 | 2.69522812 | 6.1307305 | 5.52E-09 | 4.23E-08 | 9.9629973 |
| CDKN2B | 1.07120192 | 4.30559883 | 6.10993301 | 6.14E-09 | 4.64E-08 | 9.85826063 |
| CYP1B1 | 1.29272102 | 4.64113902 | 6.10315425 | 6.36E-09 | 4.79E-08 | 9.82417253 |
| CPA3 | 1.18993217 | 4.68735883 | 6.08072293 | 7.14E-09 | 5.34E-08 | 9.71154867 |
| PTGS2 | 1.35908353 | 3.62208105 | 6.07787217 | 7.25E-09 | 5.41E-08 | 9.69725485 |
| ANO1 | 1.02929321 | 6.00338694 | 6.0325138 | 9.16E-09 | 6.66E-08 | 9.4704161 |
| FLNC | 1.15868104 | 3.57696863 | 5.95518191 | 1.36E-08 | 9.48E-08 | 9.08625871 |
| CYBB | 1.10432135 | 4.85443464 | 5.860648 | 2.20E-08 | 1.46E-07 | 8.6211334 |
| PTGIS | 1.3139138 | 4.88524323 | 5.85799022 | 2.23E-08 | 1.48E-07 | 8.60812874 |
| ITGA2 | 1.00139867 | 5.46206569 | 5.81182436 | 2.81E-08 | 1.82E-07 | 8.38287216 |
| RGS1 | 1.08961984 | 5.55630739 | 5.7920724 | 3.10E-08 | 1.99E-07 | 8.28686526 |
| MEDAG | 1.36510859 | 4.16265327 | 5.78927807 | 3.14E-08 | 2.02E-07 | 8.27330099 |
| PLN | 1.18116644 | 3.30896991 | 5.75069689 | 3.81E-08 | 2.39E-07 | 8.08647546 |
| IGF2 | 1.22790312 | 4.75550287 | 5.73124168 | 4.20E-08 | 2.60E-07 | 7.99258919 |
| GPR183 | 1.118053 | 4.56608714 | 5.72608458 | 4.31E-08 | 2.66E-07 | 7.96773878 |
| TNC | 1.24879666 | 4.72843214 | 5.71801258 | 4.49E-08 | 2.76E-07 | 7.92887315 |
| CNTN1 | 1.04739502 | 3.33200316 | 5.70654625 | 4.75E-08 | 2.90E-07 | 7.873729 |
| PRRX2 | 1.0103076 | 4.02745979 | 5.68163401 | 5.37E-08 | 3.24E-07 | 7.75418297 |
| TGM2 | 1.12084652 | 7.55569896 | 5.66462629 | 5.84E-08 | 3.50E-07 | 7.67277547 |
| C1QB | 1.03511026 | 7.92551094 | 5.66129896 | 5.94E-08 | 3.55E-07 | 7.65686893 |
| CD53 | 1.07716201 | 5.82384895 | 5.65640355 | 6.09E-08 | 3.63E-07 | 7.63347777 |
| CNN1 | 1.23369347 | 5.18957734 | 5.63976629 | 6.61E-08 | 3.90E-07 | 7.55408677 |
| CILP | 1.44248308 | 3.62257787 | 5.61671066 | 7.40E-08 | 4.31E-07 | 7.44433636 |
| LMOD1 | 1.00267051 | 4.71209401 | 5.5817209 | 8.78E-08 | 5.02E-07 | 7.27837481 |
| CST2 | 1.40600309 | 4.89456148 | 5.57855544 | 8.92E-08 | 5.09E-07 | 7.26339627 |
| SMOC2 | 1.06188869 | 5.54785868 | 5.53426591 | 1.11E-07 | 6.16E-07 | 7.05444888 |
| MRC1 | 1.19079354 | 4.58070366 | 5.51149981 | 1.24E-07 | 6.79E-07 | 6.94749951 |
| MFAP4 | 1.10495706 | 7.14073566 | 5.46435281 | 1.55E-07 | 8.31E-07 | 6.72700558 |
| AHNAK2 | 1.19671542 | 4.35556699 | 5.46130114 | 1.58E-07 | 8.43E-07 | 6.71277996 |
| MMP3 | 1.11670824 | 2.32874993 | 5.45078557 | 1.66E-07 | 8.82E-07 | 6.66380408 |
| MMP9 | 1.26425132 | 5.54010253 | 5.38410969 | 2.28E-07 | 1.17E-06 | 6.35483056 |
| IGFL3 | 1.0009366 | 1.35052046 | 5.36287178 | 2.53E-07 | 1.29E-06 | 6.25698675 |
| CCL18 | 1.46801862 | 5.09941174 | 5.32682138 | 3.00E-07 | 1.50E-06 | 6.09153855 |
| MAB21L2 | 1.15224812 | 1.78565651 | 5.30351688 | 3.35E-07 | 1.66E-06 | 5.98501474 |
| HLA-DQA1 | 1.01086726 | 5.49412232 | 5.26899831 | 3.94E-07 | 1.93E-06 | 5.82785415 |
| CDH3 | 1.15416928 | 5.20168024 | 5.23435978 | 4.64E-07 | 2.22E-06 | 5.67089824 |
| KLK6 | 1.84595205 | 5.24984969 | 5.18468673 | 5.85E-07 | 2.74E-06 | 5.44713897 |
| CCL13 | 1.14564061 | 3.17769101 | 5.11790745 | 7.97E-07 | 3.63E-06 | 5.14879818 |
| COL7A1 | 1.14841335 | 4.04447648 | 5.10511487 | 8.46E-07 | 3.83E-06 | 5.091973 |
| PTPRC | 1.06969384 | 3.89235766 | 5.08511215 | 9.27E-07 | 4.16E-06 | 5.00333164 |
| IL7R | 1.11201147 | 3.84318395 | 5.06975636 | 9.95E-07 | 4.44E-06 | 4.93545863 |
| ANKRD1 | 1.04943903 | 2.24745597 | 4.93713234 | 1.82E-06 | 7.67E-06 | 4.35566431 |
| MUC16 | 1.08545124 | 1.81394405 | 4.82408162 | 3.01E-06 | 1.21E-05 | 3.87063846 |
| SEMA7A | 1.02243756 | 5.009145 | 4.74559682 | 4.26E-06 | 1.65E-05 | 3.53897962 |
| MARCO | 1.38105778 | 4.18634284 | 4.7398671 | 4.37E-06 | 1.69E-05 | 3.51493151 |
| DKK1 | 1.34296207 | 4.00066921 | 4.64944345 | 6.47E-06 | 2.41E-05 | 3.13840522 |
| S100A9 | 1.01805968 | 6.915781 | 4.62378704 | 7.23E-06 | 2.66E-05 | 3.03260171 |
| CXCL10 | 1.0891018 | 4.04812662 | 4.5305392 | 1.08E-05 | 3.83E-05 | 2.65193809 |
| JCHAIN | 1.5045899 | 7.22339575 | 4.47793176 | 1.35E-05 | 4.68E-05 | 2.43988686 |
| LAMC2 | 1.16312993 | 7.72609641 | 4.42436034 | 1.68E-05 | 5.74E-05 | 2.22597683 |
| AREG | 1.02986938 | 5.28612142 | 4.37203277 | 2.09E-05 | 7.00E-05 | 2.01902354 |
| PTGES | 1.00094698 | 5.46291609 | 4.29449888 | 2.88E-05 | 9.31E-05 | 1.71602877 |
| SMIM32 | -1.0151845 | 2.29101433 | -4.2935076 | 2.89E-05 | 9.35E-05 | 1.71218329 |
| SCEL | 1.06224927 | 3.4366633 | 4.26794892 | 3.21E-05 | 0.0001028 | 1.61328307 |
| IGLL5 | 1.24853958 | 4.61517393 | 4.18217533 | 4.53E-05 | 0.00014086 | 1.28489709 |
| GPR87 | 1.16444683 | 2.9227511 | 4.13929049 | 5.37E-05 | 0.00016414 | 1.12275912 |
| ACTG2 | 1.21079435 | 4.37899925 | 4.08043927 | 6.78E-05 | 0.00020265 | 0.90249707 |
| ITGB6 | 1.08747512 | 6.23334099 | 4.05455602 | 7.51E-05 | 0.0002219 | 0.80644979 |
| VTN | -1.0518909 | 3.42986962 | -3.9479622 | 0.00011347 | 0.00032199 | 0.41626576 |
| CXCL9 | 1.03315818 | 4.03049823 | 3.93308356 | 0.00012013 | 0.00033958 | 0.36249365 |
| KRT17 | 1.41636887 | 8.11556021 | 3.76503193 | 0.00022619 | 0.00060354 | -0.2329497 |
| PCSK1N | -1.2611548 | 5.33981407 | -3.7629869 | 0.00022791 | 0.00060793 | -0.2400599 |
| KLK8 | 1.08750293 | 2.79609984 | 3.73009326 | 0.00025733 | 0.00067907 | -0.3539735 |
| KRT6A | 1.50202279 | 4.14328711 | 3.6945415 | 0.00029315 | 0.00076383 | -0.4761318 |
| CRABP2 | 1.04654991 | 6.59273931 | 3.67118536 | 0.00031919 | 0.00082614 | -0.5558398 |
| TRIM29 | 1.09325546 | 5.02003696 | 3.65908228 | 0.00033353 | 0.00085862 | -0.5969738 |
| KRT6B | 1.03636633 | 3.83797505 | 3.63632796 | 0.00036214 | 0.00092645 | -0.6739914 |
| CST6 | 1.20235194 | 4.71042433 | 3.62324607 | 0.00037963 | 0.00096603 | -0.7180832 |
| CXCL14 | 1.0089328 | 7.38577604 | 3.5739124 | 0.00045299 | 0.00113205 | -0.8831262 |
| KRT5 | 1.30644325 | 3.42425927 | 3.47382804 | 0.00064464 | 0.00155526 | -1.2119245 |
| GPRC5A | 1.00339527 | 7.15112454 | 3.46218062 | 0.00067133 | 0.00161536 | -1.2496608 |
| KLK7 | 1.27133617 | 4.71002747 | 3.42242419 | 0.00077044 | 0.00183303 | -1.377633 |
| LY6D | 1.36206504 | 3.53645392 | 3.37161675 | 0.00091708 | 0.00214318 | -1.5392923 |
| MMP7 | 1.11915668 | 9.1822069 | 3.30938848 | 0.0011322 | 0.00259938 | -1.7343919 |
| CXCL5 | 1.2422879 | 5.73268541 | 3.27346472 | 0.00127693 | 0.00289944 | -1.8455598 |
| PHGR1 | -1.0414189 | 3.11978346 | -3.1950893 | 0.00165446 | 0.00367085 | -2.0843608 |
| SCGB3A1 | -1.0842457 | 2.8649373 | -3.1300032 | 0.0020441 | 0.00444592 | -2.278751 |
| CST1 | 1.08709644 | 6.36429518 | 3.03202289 | 0.002793 | 0.00588419 | -2.5646209 |
| PADI1 | 1.06231716 | 3.80604386 | 2.95547793 | 0.00354577 | 0.00729769 | -2.7822478 |
| S100A2 | 1.07412635 | 5.01866458 | 2.94073215 | 0.00371062 | 0.00760437 | -2.8235936 |
| GABRP | 1.04622838 | 5.36196185 | 2.7469372 | 0.00663567 | 0.01283179 | -3.3494513 |
| PDIA2 | -1.0394758 | 3.63723546 | -2.6833091 | 0.00797885 | 0.0151245 | -3.5149403 |
| MTRNR2L1 | -1.0536138 | 2.26609043 | -2.3067211 | 0.02222493 | 0.03794734 | -4.4204941 |
| PSCA | 1.02245754 | 6.41338124 | 2.19813616 | 0.02923272 | 0.04851507 | -4.6577519 |
