# Supplementary material for: Systematic Analysis of an Invasion-Related 3-Gene Signature and Its Validation as a Prognostic Model for Pancreatic Cancer
Source: Front Oncol. 2021 Dec 15;11:759586. doi: 10.3389/fonc.2021.759586 (PMC8715959; doi:10.3389/fonc.2021.759586)
Supplement: Supplementary file 6 [file Table_4.docx]

Supplement Table 4. Comparison of prediction performance of different models

| **Models** | **1-year AUC** | **2-year AUC** | **3-year AUC** | **Survival curve** |
| --- | --- | --- | --- | --- |
| Chen | 0.74 | 0.76 | 0.78 | P = 0.00017, 95% (1.65-2.66) |
| Cheng | 0.72 | 0.68 | 0.68 | P = 0.00021, 95% (1.48-2.54) |
| Stratford | 0.61 | 0.67 | 0.73 | P = 0.00054, 95% (1.27-1.98) |
| Our model | 0.73 | 0.77 | 0.81 | P < 0.0001, 95% (1.65-2.71) |
